# Integrated screening and treatment services for HIV, hypertension and diabetes in Kenya: assessing the epidemiological impact and cost‐effectiveness from a national and regional perspective

**DOI:** 10.1002/jia2.25499

**Published:** 2020-06-19

**Authors:** Parastu Kasaie, Brian Weir, Melissa Schnure, Chen Dun, Jeff Pennington, Yu Teng, Richard Wamai, Kipkoech Mutai, David Dowdy, Chris Beyrer

**Affiliations:** ^1^ Department of Epidemiology The Johns Hopkins Bloomberg School of Public Health Baltimore MD USA; ^2^ Department of Health, Behavior and Society The Johns Hopkins Bloomberg School of Public Health Baltimore MD USA; ^3^ Avenir Health Glastonbury CT USA; ^4^ Department of Cultures, Societies and Global Studies Integrated Initiative for Global Health Northeastern University Boston MA USA; ^5^ National AIDS Control Council Nairobi Kenya

**Keywords:** HIV, diabetes mellitus, hypertension, Kenya, cost‐benefit analysis, computer simulation

## Abstract

**Introduction:**

As people with HIV age, prevention and management of other communicable and non‐communicable diseases (NCDs) will become increasingly important. Integration of screening and treatment for HIV and NCDs is a promising approach for addressing the dual burden of these diseases. The aim of this study was to assess the epidemiological impact and cost‐effectiveness of a community‐wide integrated programme for screening and treatment of HIV, hypertension and diabetes in Kenya.

**Methods:**

Coupling a microsimulation of cardiovascular diseases (CVDs) with a population‐based model of HIV dynamics (the Spectrum), we created a hybrid HIV/CVD model. Interventions were modelled from year 2019 (baseline) to 2023, and population was followed to 2033. Analyses were carried at a national level and for three selected regions (Nairobi, Coast and Central).

**Results:**

At a national level, the model projected 7.62 million individuals living with untreated hypertension, 692,000 with untreated diabetes and 592,000 individuals in need of ART in year 2018. Improving ART coverage from 68% at baseline to 88% in 2033 reduced HIV incidence by an estimated 64%. Providing NCD treatment to 50% of diagnosed cases from 2019 to 2023 and maintaining them on treatment afterwards could avert 116,000 CVD events and 43,600 CVD deaths in Kenya over the next 15 years. At a regional level, the estimated impact of expanded HIV services was highest in Nairobi region (averting 42,100 HIV infections compared to baseline) while Central region experienced the highest impact of expanded NCD treatment (with a reduction of 22,200 CVD events). The integrated HIV/NCD intervention could avert 7.76 million disability‐adjusted‐life‐years (DALYs) over 15 years at an estimated cost of $6.68 billion ($445.27 million per year), or $860.30 per DALY averted. At a cost‐effectiveness threshold of $2,010 per DALY averted, the probability of cost‐effectiveness was 0.92, ranging from 0.71 in Central to 0.92 in Nairobi region.

**Conclusions:**

Integrated screening and treatment of HIV and NCDs can be a cost‐effective and impactful approach to save lives of people with HIV in Kenya, although important variation exists at the regional level. Containing the substantial costs required for scale‐up will be critical for management of HIV and NCDs on a national scale.

## 1 INTRODUCTION

The HIV epidemic in Kenya is one of the largest globally, with an estimated 1.6 million people living with HIV (PLHIV) in 2018 [1]. According to the Joint United Nations Programme on HIV/AIDS (UNAIDS), approximately 89% of PLHIV knew their status and 75% were on treatment in 2018 [2], leaving a gap in HIV testing and treatment initiation. At the same time, the burden of non‐communicable diseases (NCDs) in Kenya is increasing. Data from a Kenya health and demographic surveillance system found that deaths due to NCDs increased from 35% of total deaths in 2003 to 45% in 2010 [3, 4]. The 2015 Kenya STEPwise approach to Surveillance (STEPS) survey reports the age‐standardized prevalence of hypertension at 24.5%, pre‐diabetes at 3.1%, and diabetes mellitus at 2.4% [4]. Importantly, only 15.6% of those with hypertension were aware of their elevated blood pressure, and among those aware, only 26.9% were on treatment [4]. For those with pre‐diabetes or diabetes mellitus, 43.7% were aware of their condition and about 20% were on treatment [4].

In response to the ongoing epidemic of HIV and increasing burden of NCDs, and realistic restrictions in health budget, policy makers require efficient approaches to resource allocation. One such approach is the community‐based, multi‐disease testing and treatment strategy used in the Sustainable East Africa Research in Community Health trial, or SEARCH [5]. This trial, based in rural Uganda and Kenya, provided integrated screening for HIV, hypertension and diabetes, and facilitated linkage to care for those in need of treatment [5]. The results suggest the intervention’s success in achieving high levels of testing coverage, linkage to HIV and NCD care and viral suppression after 1‐year [6, 7, 8]. Nevertheless, the population‐level impact and cost‐effectiveness of such interventions at a national level remain uncertain. Furthermore, regional heterogeneities in factors relating to HIV transmission, NCD burden and population demographics may result in differential effectiveness and efficiency of such programmes when implemented at a regional level. As such, we sought to model the epidemiological impact and cost‐effectiveness of an integrated programme similar to project SEARCH at a national and regional level in Kenya.

## 2 METHODS

**Figure 1:**
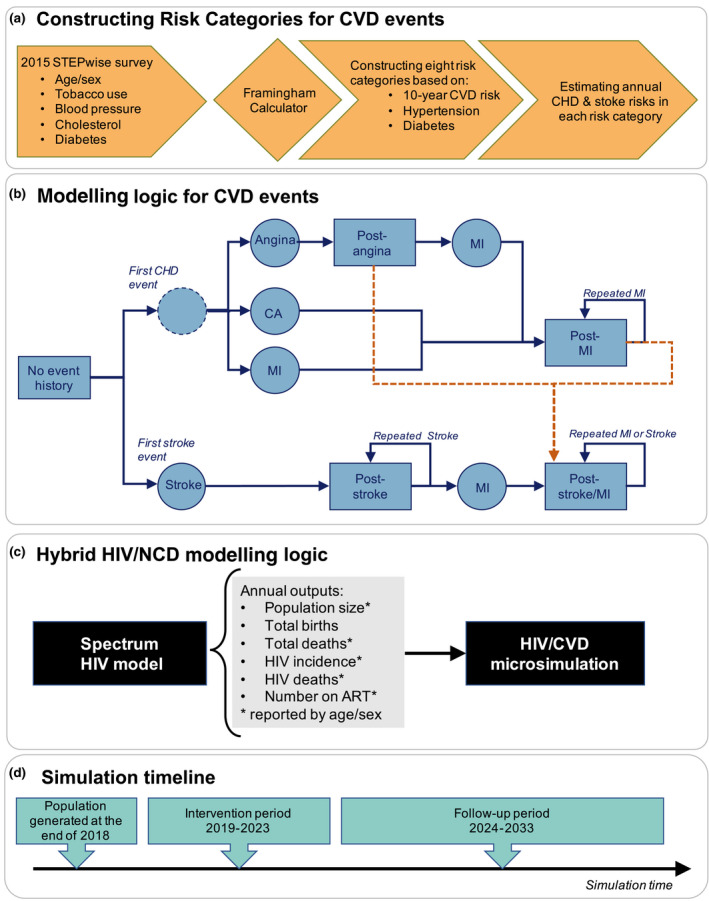
Hybrid HIV/CVD model overview. Panel A illustrates the method for defining eight cardiovascular disease (CVD) risk categories using data from the 2015 STEPwise survey in Kenya. Panel B shows the schematic model of CVD events, namely cardiac arrest (CA), angina, myocardial infarction (MI) and stroke. Following a CVD event, individuals experience a probability of acute mortality in the first year. If they survive, they subsequently move to a post‐event state in which they experience an increased annual risk of mortality, risk of new/repeated CVD events, and disability for future life years lived in the model. Dashed arrows showed in orange mark the risk of stroke among those in post‐cardiovascular heart disease (CHD) states. Panel C shows the relationships and flow of information between Spectrum and the HIV/CVD microsimulation. Panel D shows the simulation timeline, starting in year 2019 and ending in 2033. Annual outputs from the Spectrum model are used to inform the demographic processes and HIV dynamics in the HIV/CVD microsimulation. To ensure a precision of results, the baseline and intervention scenarios are modelled across 2000 random simulations. All outcomes are reported as median values across these simulations.

Coupling a microsimulation of cardiovascular diseases (CVDs) with a population‐based HIV model (the Spectrum [9]), we created a hybrid model of HIV/CVDs (Figure 1). Population demographics and HIV epidemiology were estimated from Spectrum, and individual‐level risks for NCDs were estimated from the 2015 Kenyan STEPwise survey [10]. Separate models were developed to represent Kenya at a national level and for three selected regions (namely Nairobi, Central and Coast). Access to all data and models were granted through corresponding agencies. Patient consent and ethical review were not required.

### 2.1 The Spectrum model

The Spectrum software (Avenir Health, Glastonbury, CT, USA) is applied by the Joint United Nations Programme on HIV/AIDS (UNAIDS) to estimate key HIV indicators for 161 countries around the world [9]. Country‐specific models are maintained and updated by a team of country expert on a regular basis. The Kenyan AIDS impact model (AIM) is calibrated to the 2019 official HIV estimates from National AIDS Control Council. Access to the latest release of national and regional Spectrum models was granted through UNAIDS (Data S1). This deterministic model represents a simplified representation of HIV and demographics in Kenya. This deterministic model represents a simplified representation of HIV and demographics in Kenya.

### 2.2 CVD microsimulation model

The underlying structure of our CVD microsimulation is based on a recently published cost‐effectiveness analysis of CVD management in Kenya [11]. To quantify the epidemiological and economic burden of hypertension and diabetes, we focused on the subsequent effect of these conditions on the incidence of major CVD events that could result in death or disability. For this purpose, we used the individual‐level data from the 2015 Kenyan STEPwise survey [10] and estimated the 10‐year risk of first CVD event for surveyed individuals via the Framingham calculator [12] (Figure 1A). The Framingham calculator was developed in North America and may not fully generalize to Kenya, but no other simple calculators based on African populations exist.

Next, we defined eight risk categories (Table 1) based on binary classifications of Framingham‐calculated 10‐year CVD risk (greater or less than 10%), hypertension (blood pressure greater or less than 140/90 mmHg) and Type 2 diabetes status (present or absent), and estimated the population proportion falling within each risk‐category by strata of sex and five‐year age categories (sex/age). We adjusted these estimates to match the reported prevalence of hypertension and diabetes at a national and regional level in 2015. Our underlying goal in defining these risk categories was to develop a composite measure of individual‐level risk for future CVD events—as a function of hypertension and diabetes status—which could represent the distribution of risks at a population level. Simulated individuals enter the model with an initial risk category determined according to the population risk profile. As individuals age with time, the model allows for transitions to higher risk categories.

**Table 1:** CVD risk group stratification based on 10‐year CVD risk, hypertension and diabetes status^a^

CVD risk category	Low CVD risk (<10%)^b^	Hypertension^c^	Diabetes^d^
Risk category 1	Yes	No	No
Risk category 2	No	No	No
Risk category 3	Yes	Yes	No
Risk category 4	No	Yes	No
Risk category 5	Yes	No	Yes
Risk category 6	No	No	Yes
Risk category 7	Yes	Yes	Yes
Risk category 8	No	Yes	Yes

^a^ Cardiovascular diseases (CVDs).

^b^ Ten‐year CVD risk as estimated by the Framingham calculator [12].

^c^ Individuals with systolic blood pressure ≥ 140 and/or diastolic blood pressure ≥ 90 are assumed to have hypertension.

^d^ Individuals with a plasma venous value ≥ 7.0 mmol/L (126 mg/dL) or currently on blood glucose lowering medication are assumed to have diabetes. The Kenya 2015 STEPwise approach to Surveillance (STEPS) survey data is not reported separately for type 1 and type 2 diabetes. Given the low prevalence of type 1 diabetes in Kenya (at ~ 10‐15% of total diabetes) and low prevalence of diabetes in the survey (~1.9% among both sexes), we assumed that the reported data represents type 2 diabetes.

We assumed that individuals falling within each risk category experience certain probabilities of future CVD events related to cardiovascular heart disease (CHD) (cardiac arrest, myocardial infarction or angina) and stroke. The risk of first CHD and stroke for each age, sex and ten‐year risk stratum was derived from corresponding Framingham calculators [13, 14]. Effective treatment for hypertension and diabetes are assumed to reduce the risk of both initial and subsequent CHD and stroke events; we thus conservatively ignore the benefits of such treatment on other events such as microvascular complications of diabetes. Table 2 provides a list of modelling parameters (Data S1).

**Table 2:** CVD microsimulation model parameters^a^

Parameter	Value	Reference
CVD natural history^b^		
Probability of first CHD event type		
Cardiac arrest	10%	
MI	32% (males)/ 20% (females)	[23]
Angina	1 ‐ probability of other events	[24]
Acute (one‐year) mortality following a CVD event		
Cardiac arrest	0.95	[25]
MI	0.05	[26]
Angina	0.045	[27]
Stroke	0.38	[28]
Annual mortality (post‐event)		
MI	0.04	[29]
Angina	0.03	[29]
Stroke	0.05	[29]
Annual risk of new event in post‐event states		
Repeated MI post‐MI	0.064	[30]
MI post‐angina	0.035	[31]
Repeated stroke post‐stroke	0.04	[32]
Intervention characteristics^c^		
Annual screening coverage	20% of population	Assumption
Screening success	90%	[16]
NCD treatment uptake & long‐term medication management	50%	Assumption
NCD treatment effectiveness^c^, ^d^		
% reduction in CHD events with hypertension treatment among people		[18]
‐ With diabetes	88%	
‐ Without diabetes	77%	
% reduction in stroke events with hypertension treatment among people		[18]
‐ With diabetes	74%	
‐ Without diabetes	74%	
% reduction in CHD and stroke events with diabetes treatment	79%	[19]
Costs (2018 USD)^c^		
Acute care for cardiac arrest	1,049.14	[33]
Acute care for MI	2,041.02	[33]
Acute care for angina	1,264.90	[33]
Acute care for stroke	1,916.26	[33]
Non‐acute care post‐CHD	331.15 per year	[34]
Non‐acute care post‐stroke	993.44 per year	[34]
Screening for HIV	21.50	[7]
Screening for hypertension and diabetes	1.22	[7]
HIV treatment (ART)	297.51 per year	[35]
Hypertension treatment	77.65 per year	[33]
Diabetes treatment	186.73 per year	[33]
Disability weights^c^		
Angina	0.08	[36]
Cardiac arrest	0.08	
MI	0.08 (first year) 0.072 (subsequent years)	
Stroke	0.152	
HIV	0.078 (on ART) 0.274 (off ART)	

^a^ Non‐communicable diseases (NCD); Cardiovascular diseases (CVDs); Cardiovascular heart disease (CHD); Myocardial infarction (MI).

^b^ The underlying structure of CVD natural history model and selected parameter values are based on Subramanian et al. (2019) [11].

^c^ Parameters are varied within +/‐ 15% of their original values in sensitivity analysis.

^d^ NCD treatment effectiveness is estimated separately for hypertension and diabetes treatment, and the impact of combined treatments is modelled as independent and multiplicative (Data S1).

### 2.3 Hybrid HIV/CVD model

Figure 1C shows the logical relationship and flow of information within the hybrid HIV/CVD model. The microsimulation is coded in C++, and runs in discrete time steps representing one year. Main outcomes are reported as median values and 95% uncertainty ranges across 2,000 random simulations. Due to limited space, the results are presented in terms of median values throughout the text and 95% uncertainty ranges (when available) are provided in corresponding tables in each section.

The initial population is generated according to outputs from the Spectrum model at the end of year 2018, and is characterized in terms of HIV prevalence and ART coverage by sex and age (reported as five‐year age categories). The microsimulation starts in year 2019 and runs to 2033.

Annual CVD dynamics are modelled directly at the individual‐level as described above. HIV dynamics are modelled at a population‐level via the Spectrum model, and the projected annual number of new HIV infections and HIV‐related deaths by sex/age is imported into the hybrid model. Finally, the additional probability of death due to non‐HIV/non‐CVD causes is estimated by subtracting the simulated number of HIV and CVD deaths from the projected number of all‐cause deaths in Spectrum and dividing by the population size in each sex/age stratum in each year (Data S1).

### 2.4 HIV/CVD Regional analysis

We expanded our national model of HIV/CVDs in Kenya to represent three regions including Nairobi, Coast and Central (Data S1). Each regional model was informed using estimates from the corresponding Spectrum model as discussed above. Given the lack of estimates from AIM on the impact of HIV interventions at the regional level, we used the national Spectrum model to estimate intervention’s impact. We then applied these estimates to the regional models, weighted by sex/age, to project the future size of the HIV epidemic and the number of HIV‐related deaths in each region.

### 2.5 Modelled intervention

Following the framework of project SEARCH [15], we modelled a joint community outreach campaign for screening and treatment of HIV, hypertension and diabetes over five consecutive years (2019–2023), and followed the population for a decade after the end of the intervention to year 2033. To reflect ambitious but potentially feasible implementation, we assumed that the intervention targets 20% of the population in geographically distinct communities on an annual basis, and can reach up to 90% of eligible adults (aged 15+) who are assumed to undergo screening for HIV, hypertension and diabetes [16]. The average ART uptake (i.e. accounting for loss to care and re‐engagement in care) was estimated at 73% for those HIV‐infected individuals with no previous/current ART use [5, 17].

To determine the impact of treatment for hypertension and diabetes, we estimated the average decrease in risk of future CVD events provided by long‐term treatment. Specifically, we assumed that treatment for hypertension would result in an average 10‐mmHg reduction in systolic blood pressure [18], and that treatment for diabetes would consist of metformin, a widely available and inexpensive first‐line treatment for diabetes [19] (Table 2). At baseline, we assumed that 50% of all individuals newly diagnosed with hypertension or diabetes would remain consistently in care (i.e. that the intervention would meet 50% of the total time needed to successfully complete NCD treatment); this number was assumed to incorporate both incomplete linkage to care and net losses to follow‐up (disengagement minus re‐engagement) over time and was varied in sensitivity analysis.

The intervention scenario was compared against a baseline model in which ART coverage in 2018 (final year of data) was maintained at a fixed level from 2019 to 2033, and lifetime medication management for NCDs was kept to minimal levels [20, 21, 22] (Data S1).

### 2.6 Cost‐effectiveness analysis

Cost analyses from the SEARCH study provided estimates of disease screening and ART treatment [7, 37]. Costs of standard care for CVD‐related events were based on those estimated from public/semi‐public healthcare facilities in Kenya [11]. All healthcare costs were reported in the year in which they occurred. For each scenario, disability‐adjusted life years (DALYs) were estimated as years lost to HIV‐ or CVD‐related disability and years of life lost to premature mortality. The cost‐effectiveness ratio was reported at the end of simulation period (year 2033) and was assessed against a threshold of $2,010 corresponding to Kenya’s 2019 per‐capita gross domestic product (GDP) [38]. In addition, we considered alternative cost thresholds (at increments of $500 per DALY) that reflect more stringent willingness‐to‐pay thresholds for health interventions (Data S1).

### 2.7 Sensitivity analysis

One‐way sensitivity analysis was performed by varying the value of selected parameters to +/‐15% of the original values (Table 2). For each analysis, we evaluated the changes in main outcomes in the national model, comparing integrated HIV/NCD scenario to the baseline (status‐quo) scenario.

## 3 RESULTS

### 3.1 Population summary

The simulated populations were generated based on projections from Spectrum at a national‐ and regional level in 2018 (Table 3). The national Spectrum model estimated a population size of 50.9 million, an HIV prevalence of 3.3% (2.4% among men and 4.3% among women) and ART coverage of 68% among those in need of ART. This translated to an HIV incidence of 0.97 per 1000 person‐years, corresponding to 16,419 and 30,020 new infections among men and women in 2018. The regional models reflected known heterogeneities in the burden of HIV and NCDs at local level, with the highest HIV incidence (1.09 per 1000 person‐years) in Nairobi region and the highest hypertension prevalence (37.5%) in the Central region.

**Table 3:** Baseline simulated population

Simulated population in 2018	National	Nairobi region	Central region	Coast region
Population size^a^	51.0 million	4.9 million	4.3 million	5.1 million
HIV prevalence^a^	3.35%	4.23%	3.19%	2.65%
ART coverage^a^	68.26%	67.88%	63.71%	68.71%
HIV incidence (per 1000/year)^a^	0.97	1.09	1.06	0.66
Hypertension prevalence^b^	24.1%	14.1%	37.5%	19.8%
Diabetes prevalence^b^	2.13%	3.84%	2.71%	2.01%

^a^ Population size and HIV outcomes were projected by the national and regional Spectrum AIDS Impact Models (AIM), calibrated to the 2019 official HIV estimates from national AIDS control council. Given the deterministic nature of the model, no information is available on uncertainty ranges.

^b^ The baseline prevalence of hypertension and diabetes in each model were calibrated to estimated values from the Kenya 2015 STEPwise approach to Surveillance (STEPS) survey respectively. The prevalence is shown for individuals between the ages of 15 to 70 years, similar to the STEP survey. Values represent the median values across 2,000 simulations (uncertainty ranges were too small to show).

### 3.2 HIV‐related outcomes

In the status‐quo scenario (maintaining ART coverage to reported levels at the end of year 2018), the HIV incidence in Kenya was projected to fall by 26% (ranging from 22% in Coast to 26% in Nairobi region) from 2019 to 2033 (Table 4). Increasing ART coverage to 88% of all people diagnosed with HIV by 2033 resulted in HIV incidence to fall by 64% within the same period. Compared to baseline, this corresponded to averting 347,000 HIV infections and 289,000 HIV deaths in Kenya by 2033. At a regional level, the absolute impact of expanded HIV services was highest in Nairobi, averting 42,000 HIV infections and 37,000 HIV deaths compared to baseline. However, the efficiency of expanded ART was highest in Coast region, with 0.06 HIV infections averted per additional person‐year on ART. Despite the large reductions in HIV incidence and mortality under expanded ART, HIV prevalence remained relatively stable, reflecting better survival among individuals consistently on ART.

**Table 4:** Summary of HIV‐related outcomes in Kenya^a^

	National		Nairobi region		Central region		Coast region	
	Baseline	Intervention	Baseline	Intervention	Baseline	Intervention	Baseline	Intervention
ART coverage								
Proportion of people living with HIV on ART in 2033	68.26%	88.21%	67.88%	87.39%	68.71%	87.50%	63.71%	85.75%
Total number on ART from 2019 to 2033 (million)	37.46 [32.20 to 53.10]	45.86 [38.3 to 62.70]	4.87 [4.20 to 6.90]	5.94 [5.00 to 8.10]	2.78 [2.40 to 3.90]	3.36 [2.80 to 4.60]	2.98 [2.60 to 4.20]	3.56 [3.00 to 4.90]
Additional person year on ART (million)		8.40 [6.20 to 10.10]		1.07 [0.80 to 1.30]		0.58 [0.40 to 0.70]		0.58 [0.40 to 0.70]
HIV incidence (per 1000/year)								
2018	0.97 [0.59 to 1.68]		1.09 [0.67 to 1.89]		0.66 [0.40 to 1.14]		1.06 [0.65 to 1.84]	
2033	0.72 [0.52 to 1.37]	0.35 [0.28 to 0.59]	0.81 [0.59 to 1.54]	0.39 [0.31 to 0.65]	0.51 [0.37 to 0.97]	0.25 [0.20 to 0.42]	0.83 [0.60 to 1.58]	0.39 [0.31 to 0.65]
% Reduction	25.77% [7.10% to 45.48%]	63.92% [48.29% to 66.06%]	25.69% [7.07% to 45.33%]	64.22% [48.52% to 66.37%]	22.73% [6.26% to 40.11%]	62.12% [46.93% to 64.20%]	21.7% [5.98% to 38.29%]	63.21% [47.76% to 65.32%]
HIV prevalence								
2018	3.35% [2.87% to 4.26%]		4.23% [3.62% to 5.37%]		2.65% [2.27% to 3.37%]		3.19% [2.73% to 4.05%]	
2033	2.43% [1.80% to 3.78%]	2.33% [1.73% to 3.62%]	2.99% [2.22% to 4.65%]	2.88% [2.14% to 4.48%]	2.03% [1.51% to 3.16%]	1.96% [1.45% to 3.05%]	2.37% [1.76% to 3.69%]	2.15% [1.60% to 3.34%]
% Reduction	27.55% [20.50% to 30.62%]	30.39% [26.58% to 32.33%]	29.35% [21.84% to 32.62%]	31.89% [27.89% to 33.93%]	23.41% [17.42% to 26.01%]	25.99% [22.73% to 27.65%]	25.54% [19.00% to 28.28%]	32.53% [28.45% to 34.61%]
New HIV infections								
Total from 2019 to 2033 (thousands)	734.27 [521.75 to 1,261.79]	387.55 [317.42 to 637.93]	87.01 [61.82 to 149.52]	44.87 [36.75 to 73.85]	49.45 [35.13 to 84.97]	25.7 [21.05 to 42.30]	71.28 [50.64 to 122.49]	36.56 [29.94 to 60.18]
Infections averted (thousands)		346.73 [196.79 to 629.64]		42.15 [23.92 to 76.54]		23.76 [13.48 to 43.15]		34.72 [19.70 to 63.05]
Infections averted per additional ART person/year		0.041		0.039		0.041		0.06
HIV deaths								
Total from 2019 to 2033 (thousands)	544.61 [449.49 to 619.60]	255.86 [217.87 to 295.29]	69.54 [57.39 to 79.11]	32.82 [27.94 to 37.88]	41.76 [34.46 to 48.51]	21.77 [18.53 to 25.12]	45.13 [37.24 to 51.34]	23.91 [20.36 to 27.59]
Deaths averted (thousands)		288.76 [231.79 to 342.25]		36.72 [29.47 to 43.52]		19.99 [16.04 to 23.69]		21.22 [17.03 to 25.15]
Deaths averted per additional ART person/year		0.034		0.034		0.034		0.036

^a^ HIV‐related outcomes are projected by the Spectrum model for the baseline and intervention at a national and regional level. Models are initialized with a similar population in 2018 and are followed to year 2033. The baseline scenario assumes a fixed ART coverage at 2018 levels over time. The intervention scenario models a gradual increase in coverage of ART from 2019 to 2023 [assuming a fixed coverage afterwards, from year 2024 to 2033). Values represents the median value [95% uncertainty ranges]. Uncertainty ranges are estimated across 1000 random simulations (generated by permuting epidemiological and behaviour parameters), weighted and resampled based on goodness of fit to the historical prevalence data(see Section 1.4 in Data S1).

### 3.3 NCD‐related outcomes

**Figure 2:**
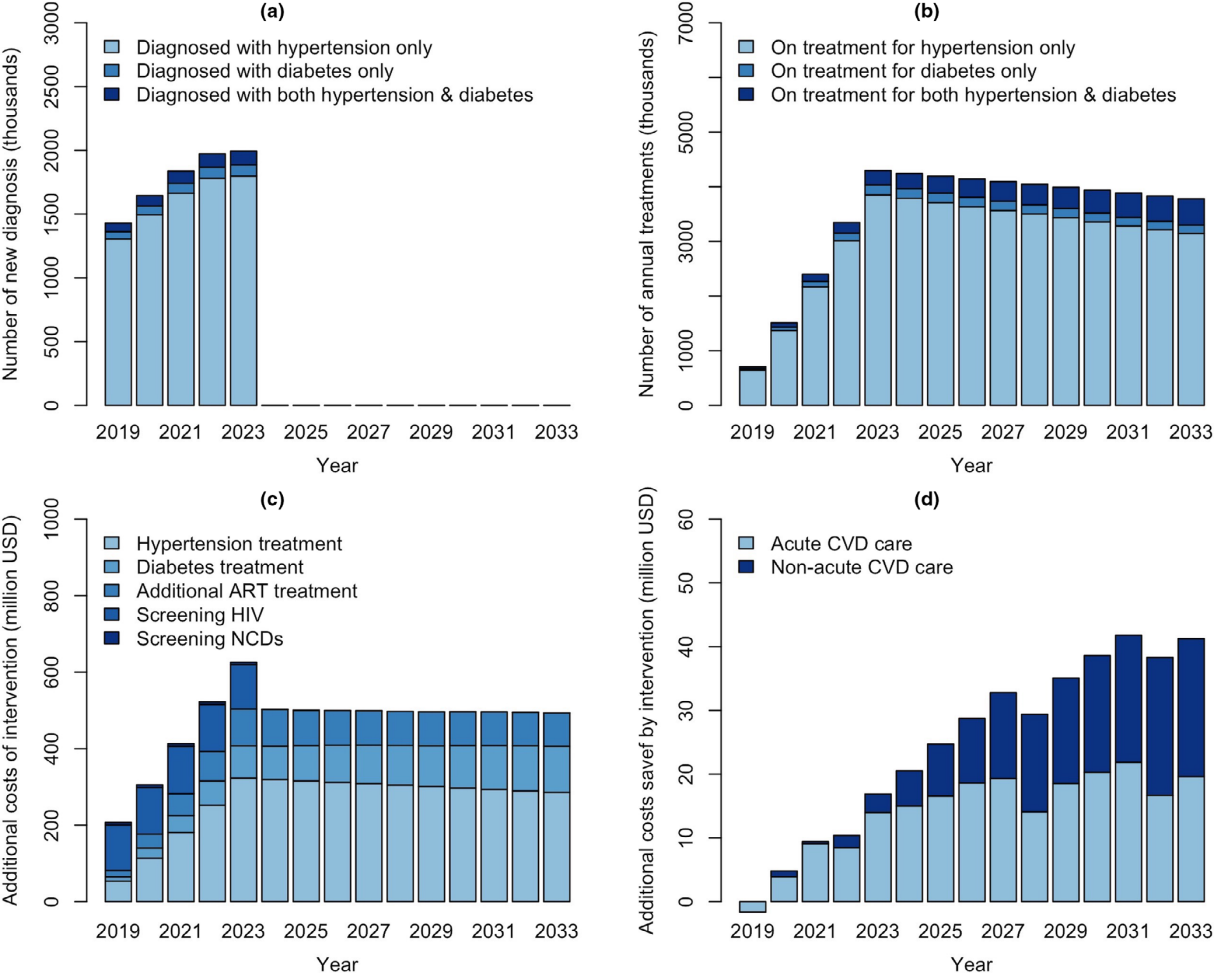
Projected outcomes from combined HIV/NCD diagnosis and management in Kenya. The intervention runs from 2019‐2023, screening 20% of the population on an annual basis for HIV, hypertension and diabetes. Panel A shows the annual number of people diagnosed with hypertension and/or diabetes. The intervention further provides treatment to a proportion of those diagnosed with HIV, hypertension and/or diabetes. Panel B shows the number of individuals receiving treatment for hypertension and/or diabetes over time. Costs are divided into two groups, including additional costs required for disease screening and treatment (Panel C) and costs saved by averting future CVD events (Panel D). ART, antiretroviral therapy; NCD, non‐communicable diseases; CVD, cardiovascular disease.

At a national level and in the absence of expanded treatment for HIV, Diabetes and Hypertension, the model projected the prevalence of untreated hypertension and diabetes at 32.43% and 4.27% respectively by 2033 (Table 5). The HIV/NCD integrated screening covered estimated 5.5 million individuals annually from 2019 to 2023, diagnosing over 8.5m individuals with hypertension and 0.83 m with diabetes (Figure 2A). Assuming that these diagnoses result in treatment for 50% of subsequent eligible treatment time, the intervention was projected to reduce the prevalence of untreated hypertension to 27.51% and of untreated diabetes to 3.42% by 2033. This resulted in averting 116,600 CVD events and 43,600 CVD deaths by 2033. At a regional level, the intervention impact was highest in the Central region, averting 22,200 CVD events and 8300 CVD deaths over the next 15 years.

**Table 5:** Summary of NCD‐related outcomes in Kenya^a^, ^b^

	National	Nairobi region	Central region	Coast region
NCDs prevalence in 2033^c^				
Baseline:				
Untreated hypertension	32.43% [32.40% to 32.45%]	25.94% [25.91% to 25.97%]	47.89% [47.86% to 47.91%]	26.41% [26.39% to 26.44%]
Untreated diabetes	4.27% [4.25% to 4.28%]	9.75% [9.73% to 9.77%]	6.05% [6.04% to 6.07%]	3.4% [3.39% to 3.42%]
Intervention:				
Untreated hypertension	27.51% [27.48% to 27.53%]	22.61% [22.58% to 22.63%]	38.73% [38.7% to 38.76%]	22.58% [22.55% to 22.61%]
Untreated diabetes	3.42% [3.41% to 3.44%]	8.43% [8.41% to 8.45%]	4.43% [4.42% to 4.45%]	2.79% [2.78% to 2.8%]
CVD events averted (2019 to 2033)				
MI	24,900 [17,900 to 31,300]	1600 [1000 to 2300]	4900 [4,100 to 5,700]	2000 [1,400 to 2,600]
Angina	12,500 [7,800 to 17,000]	1200 [700 to 1600]	3000 [2400 to 3500]	1100 [700 to 1500]
Cardiac Arrest	3200 [1200 to 5100]	200 [0 to 400]	500 [300 to 800]	200 [0 to 400]
Stroke	76,000 [66,400 to 86,100]	6200 [5300 to 7200]	13,800 [12,500 to 14,900]	5300 [4300 to 6200]
Total	116,600 [104,300 to 128,300]	9200 [8100 to 10,300]	22,200 [20,600 to 23,700]	8600 [7500 to 9800]
CVD deaths averted (2019 to 2033)				
MI	4800 [2200 to 7500]	300 [0 to 600]	1000 [700 to 1300]	1000 [700 to 1300]
Angina	1,500 [−700 to 3700]	100 [−100 to 400]	400 [200 to 700]	400 [200 to 700]
Cardiac Arrest	3000 [1000 to 4800]	200 [0 to 400]	500 [300 to 700]	500 [300 to 700]
Stroke	34,200 [27,700 to 41,000]	2800 [2100 to 3400]	6400 [5600 to 7200]	6400 [5600 to 7200]
Total	43,600 [3,6400 to 50,400]	3400 [2700 to 4100]	8300 [7500 to 9100]	8300 [7500 to 9100]

^a^ Non‐communicable diseases (NCD); Cardiovascular disease (CVD); Myocardial infarction (MI).

^b^ NCD‐related outcomes are projected by the HIV/NCD microsimulation for the baseline and intervention at a national and regional level. Models are initialized with a similar population in 2018 and are followed to year 2033. The baseline scenario assumes minimal NCD treatment. The intervention scenario models an annual campaign for screening and treatment on NCDs running from 2019‐2023. Values represents the median [95% uncertainty ranges] across 2000 random simulations.

^c^ See Data S1 for the uncertainty around the number people with untreated diabetes and hypertension in the initial and final cohort.

### 3.4 Cost‐effectiveness analysis

**Figure 3:**
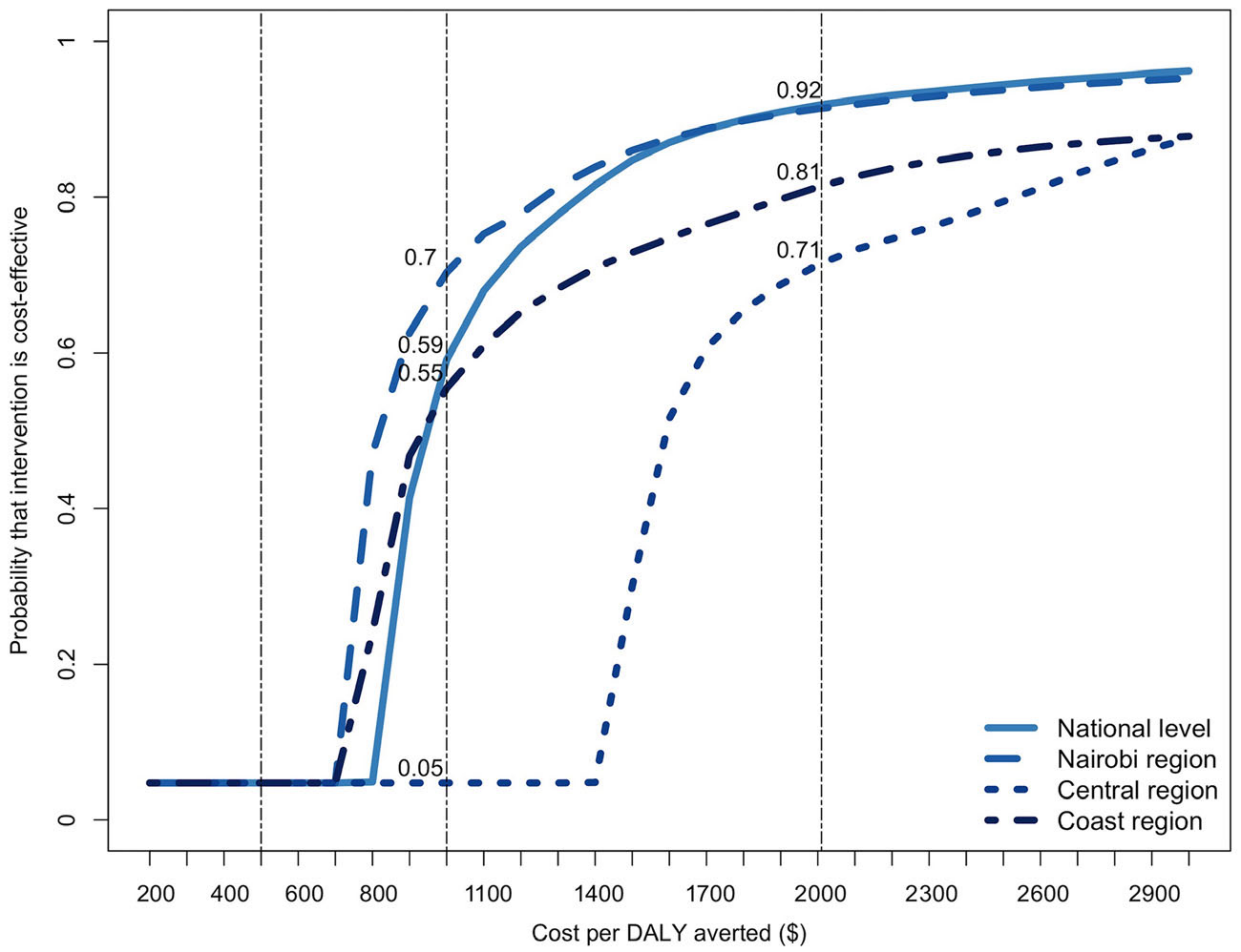
Cost‐effectiveness acceptability curves for integrated HIV and NCD diagnosis and management in Kenya. The x‐axis shows the cost per disability‐adjusted life year (DALY) averted by the intervention, and the y‐axis shows the proportion of stochastic simulations falling below the corresponding cost‐effectiveness threshold. Vertical lines represent alternative thresholds for evaluating cost‐effectiveness at $500, $1000 and $2010 (Kenya’s 2019 per‐capita gross domestic product). NCD, non‐communicable diseases.

At a national level, the incremental costs of HIV/NCD integrated programme was estimated at $6.68b over 15 years (Table 6). This reflected additional costs needed for: 5 years of screening for HIV ($0.6b) and hypertension and diabetes ($0.03b); increased treatment costs for HIV ($1.18b), diabetes ($1.28b) and hypertension ($3.95b); as well as costs saved for CVD care ($‐0.35b) (Table 6; Figure 3). The intervention was estimated to avert 7.76m DALYs, for an incremental cost‐effectiveness ratio of $860 per DALY averted at the national‐level. At a regional level, the intervention resulted in highest incremental costs in the Central region ($945m) and saved the most DALYs in Nairobi (840,000). The cost per DALY averted was $754 in Nairobi, $818 in the Coastal region, and $1500 in the Central region, all below the per capita GDP of $2010 in Kenya.

**Table 6:** Costs and DALYs required/saved by the integrated HIV and NCD care in Kenya^a^, ^b^

	National	Nairobi region	Central region	Coast region
Saved costs (2018 US dollars)				
Acute CVD care	0.22 [0.19 to 0.24] billion	16.94 [14.77 to 19.01] million	40.70 [37.72 to 43.45] million	15.85 [13.64 to 18.02] million
Non‐acute CVD care	0.15 [0.11 to 0.20] billion	14.33 [10.43 to 18.56] million	32.18 [26.82 to 37.56] million	11.08 [7.02 to 18.02] million
Additional costs (2018 US dollars)				
ART	1.18 [1.17 to 1.20] billion	133.71 [132.20 to 135.16] million	83.81 [82.45 to 85.13] million	91.16 [89.83 to 92.51] million
Diabetes treatment	1.28 [1.27 to 1.29] billion	211.9 [210.67 to 213.23] million	207.46 [206.27 to 208.58] million	84.18 [83.36 to 84.98] million
Hypertension treatment				
Screening for HIV	603.34 [603.12 to 603.58] million	62.03 [62.00 to 62.05] million	67.63 [67.61 to 67.65] million	50.56 [50.54 to 50.59] million
Screening for diabetes and hypertension	34.24 [34.22 to 34.25] million	3,519.73 [3,521.09 to 3,518.35] thousand	3,837.83 [3,839.01 to 3,836.62] thousand	2,869.06 [2,870.42 to 2,867.70] thousand
Total Costs (2018 US dollars)				
Incremental costs	6.68 [6.61 to 6.74] billion	632.95 [626.79 to 638.75] million	945.29 [937.44 to 93.05] million	471.59 [466.08 to 476.99] million
Total DALYs				
Incremental DALYs averted	7.76 [8.01 to 7.51] million	839.13 [811.93 to 865.24]	632.88 [608.74 to 657.06]	576.82 [547.86 to 604.93]
Incremental costs per DALY (2018 USD)				
	860.36 [830.59 to 890.66]	754.04 [729.63 to 780.26]	1,493.07 [1,435.63 to 1,555.65]	818.01 [864.38 to 776.50]

^a^ Non‐communicable diseases (NCD); Disability‐adjusted life year (DALY); Cardiovascular disease (CVD).

^b^ Values represent the differences in simulated costs and DALYs between the baseline and intervention at a national and regional level. Future costs are discounted at 3%. Values represents the median [95% uncertainty ranges] across 2,000 random simulations.

Using the threshold of $2010 per DALY averted, the probability that the intervention would be cost‐effective was 91.8% at a national level, ranging from 71.28% in Central to 91.35% in Nairobi. As policy makers may prefer different willingness‐to‐pay thresholds, [39] we explored other thresholds through sensitivity analyses (Figure 3). Lowering the willingness‐to‐pay threshold to $1,000 reduced the probability of cost‐effectiveness to 59% at a national level, while increasing the heterogeneity at a regional level (with probabilities ranging from 70% in Nairobi to 55% in Coast and only 5% in the Central region). The intervention did not remain cost‐effective under a threshold $500.

### 3.5 Sensitivity analysis

**Figure 4:**
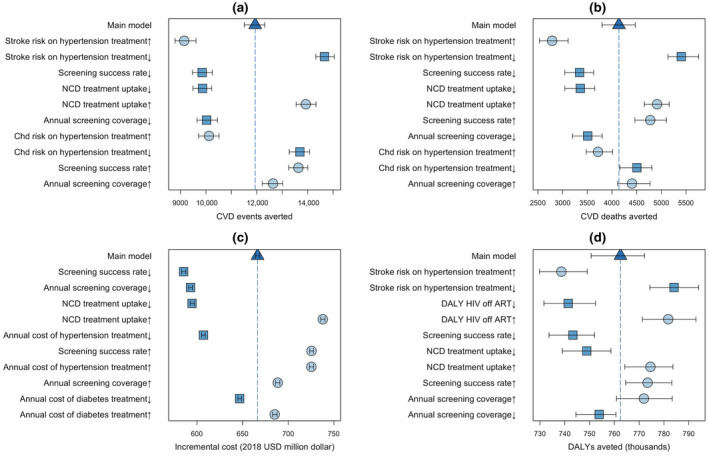
One‐way sensitivity analysis to value of selected model parameters. Panels show the sensitivity of epidemiological outputs, including the number of CVD events (panel A) and deaths (panel B) averted, and costing outcomes, including the incremental cost of intervention (panel C) and DALYs averted (panel D), under one‐way variation in the value of selected model parameters. Each parameter value is followed by an up/down arrow, denoting a 15% increase (circle marks) or decrease (square marks) in the input parameter value as listed in Table 3. Each scenario is simulated starting in year 2019 and is followed to year 2033. The bars and arrows represent the median and interquartile ranges across 500 simulations. The triangle mark and dashed line represent the main model with no parameter variation. The results are summarized by showing the ten parameters for which variation resulted in the largest variations from the main model (decreasing impact from top to bottom. CVD, cardiovascular disease; NCD, non‐communicable diseases; DALY, disability‐adjusted life year; ART, antiretroviral therapy.

All outcomes were sensitive to variation in value of parameters related to NCD screening/treatment coverage (e.g. annual screening coverage, screening success rate and NCD treatment uptake) (Figure 4). The epidemiological impact of intervention (measured by the number of CVD events and deaths averted) was also sensitive to variation in NCD treatment effectiveness (modelled as reduction in risk of CHD and stroke). The incremental costs were also sensitive to variation in cost of hypertension and diabetes treatment, comprising the biggest portion of intervention costs. With < 4% variations, DALYs were robust to variation in value of selected parameters, suggesting that the majority of DALYs in this population was due to HIV infection.

## 4 DISCUSSION

Integrated, population‐based screening and treatment for HIV and NCDs in Kenya could have substantial impact over 15 years, averting 64% of new HIV infections, 284,000 HIV‐related deaths, 43,600 CVD‐related deaths, and 7.8 million HIV‐ and CVD‐related DALYs. At a commonly used threshold for cost‐effectiveness (less than per capita GDP per DALY averted), this intervention was more than 90% likely to be cost‐effective. However, the cost required to fully scale up this intervention was substantial, with a 15‐year incremental cost of $6.7 billion dollars, equivalent to an increase of 12% in Kenya’s total health budget [40]. These results illustrate that integrated HIV/NCD diagnosis and management has the potential to be highly impactful and moderately cost‐effective in a country like Kenya, but achieving these gains will only be possible with sustained financial and political commitment.

Our regional models reveal important geographic variation. Compared to the national estimate, the cost per DALY averted (for HIV and NCDs) was 74% higher in the Central region, where the prevalence was higher for hypertension but lower for HIV. These results are in line with previous reports of health disparities across counties in Kenya [41]. In scaling up integrated HIV/NCD care, therefore, the most efficient use of resources may be to first focus integration efforts on those regions with higher HIV prevalence, while maintaining separate HIV and NCD systems (i.e. focusing NCD management on older and other high‐risk populations [42]) in settings where NCD prevalence is high but HIV prevalence is low.

Both overall costs and cost‐effectiveness were highly sensitive to the cost of hypertension management, owing to the high prevalence of hypertension in this population (ranging from 2.3 times the prevalence of HIV in Nairobi region to 7.6 times HIV prevalence in Central region). We assumed that management of hypertension would cost $78 per person‐year, similar to other public‐sector studies in sub‐Saharan Africa [33, 42] but lower than at least one estimate from Oyando et al. ($304 per person‐year)[43]. In a study of five rural counties in Western Kenya, Osetinsky et al. [44] find the cost of chronic disease medicine programmes to be lowest compared to other public NCD care programmes. However, the estimated per patient annual cost of NCD care in these programmes ($27.50 to $154.06) was still higher than the per capita healthcare budget contributions ($17.50 to $20.00) in these counties, suggesting a large gap in budget for expanding and sustaining NCD care even via most efficient care delivery models. In our study, the costs of hypertension management accounted for over 60% of total costs, but CVD deaths accounted for only 15% of all deaths averted; thus, at this cost, HIV care appears much more cost‐effective than NCD management. Mounting a national effort to manage hypertension and diabetes may hinge on cost minimization, which may include strategies from the HIV response where the cost of first‐line HIV medications decreased by 99% from $10,000 per person in 2000 to $116 per person in 2010 [45, 46].

As with any modelling study, this analysis has important limitations. First, both Spectrum and our NCD model employ a number of simplifying assumptions (e.g. heterogeneous mixing within risk groups, estimating region‐level impact of HIV interventions as reflective of national‐level estimates, CVD risk within fixed categories) that do not fully capture the complex interplay between HIV and NCDs in Kenya. Second, we estimated CVD risk using the Framingham calculator, which allows for simple estimation of risk with a small number of input parameters and was calibrated to a largely white male population and may not accurately estimate CVD risk in African populations [47]. As genetics also play a role in NCD risks and Africa has genetically and ethnically diverse populations [48, 49], further studies (e.g. using potential electronic health records) determine better prediction models for NCD‐risk‐profiles in African populations. Third, by focusing only on stroke and major CHD events, our model ignores other positive benefits of treating hypertension and diabetes (for example, reductions in microvascular complications of diabetes). Thus, our estimates of DALYs averted may be conservative. Fourth, in the absence of accepted cost‐effectiveness thresholds, we benchmarked cost‐effectiveness primarily against Kenya’s per‐capita GDP. Recent arguments [50] suggest that per‐capita GDP thresholds may result in labelling a number of unaffordable interventions as “cost‐effective”; to the extent that Kenya’s true willingness or ability to pay for health interventions is lower, our model may be overly optimistic in its estimates of cost‐effectiveness. Fifth, we did not account for dynamic changes in CVD risk (within sex/age stratum) over time. As trends in smoking and dietary intake change in Kenya [51, 52, 53, 54], our estimates of future CVD risk may be underestimated or overestimated. Finally, some of our estimates (e.g. estimated population in specific low‐probability CVD risk categories, particularly at the regional‐level) are based on small sample sizes and thus subject to substantial uncertainty.

## 5 CONCLUSIONS

We have constructed a model to evaluate the likely population‐level impact and cost‐effectiveness of a potential integrated HIV/NCD diagnosis and management programme based on that of Project SEARCH in Kenya. We find that such an integrated programme could save more than 300,000 lives over a 15‐year period, with substantial improvements in the dual epidemics of HIV and NCDs in this setting. Such a programme could be moderately cost‐effective; cost‐effectiveness could be substantially improved by lowering the cost of hypertension management. Important variations also existed at the subnational‐level, arguing for a targeted approach to HIV/NCD integration that first focuses on regions with higher HIV prevalence. These findings are likely to generalize to other East African countries with similar epidemiology and economic conditions and may inform intervention design in sub‐Saharan Africa more broadly, though studies of specific interventions in specific contexts are needed. Achieving these gains will only be possible with sufficient political will and ensuring increased financial commitment to support scale‐up at the regional and national‐levels.

Two decades ago, researchers had shown the epidemiological basis for CVDs control policies in sub‐Saharan Africa [55]. Given Kenya’s 2030 Health Policy framework and Big 4 Agenda for expanding universal health coverage [56, 57], studies showing health insurance to have a positive effect on stemming HIV [58], and increasing affordability for NCDs treatments [33], studies like ours are useful in advancing such national polices. Results from our modelling study in Kenya emphasize the critical need for an integrated approach to tackle the growing burden of HIV and NCDs over the next decade.

## COMPETING INTERESTS

The authors declare that they have no competing interests.

## AUTHORS’ CONTRIBUTIONS

PK, BW, DD and CB contributed to conception and design. PK, MS, CD, JP and YT contributed to model development. PK, MS, CD and YT contributed to analysis. PK, BW, RW, KM, DD and CB contributed to interpretation and important intellectual input. PK, MS and DD contributed to first draft of manuscript. PK, BW, MS, CD, JP, YT, RW, KM, DD and CB contributed to manuscript review and revision. All authors have read and approved the final manuscript.

## Supporting information

Data S1: Supporting information and additional results
