# Supplementary material for: Integrated screening and treatment services for HIV, hypertension and diabetes in Kenya: assessing the epidemiological impact and cost‐effectiveness from a national and regional perspective
Source: J Int AIDS Soc. 2020 Jun 19;23(Suppl 1):e25499. doi: 10.1002/jia2.25499 (PMC7305418; doi:10.1002/jia2.25499)
Supplement: Supplementary file 1 — Data S1: Supporting information and additional results [file JIA2-23-e25499-s001.docx]

**Supplementary material**

**Integrated screening and treatment services for HIV, hypertension and diabetes in Kenya: assessing the epidemiological impact and cost-effectiveness from a national and regional perspective**

Authors:

Parastu Kasaie, Brian Weir, Melissa Schnure, Chen Dun, Jeff Pennington, Yu Teng, Richard Wamai, Kipkoech Mutai, David Dowdy, and Chris Beyrer

# The SPECTRUM model:

The SPECTRUM (<https://www.avenirhealth.org/software-spectrum.php>) is a suite of easy to use models which provides policy makers with an analytical tool to support decision making. The models are well known for their formal applications by UNIAIDS to estimate the national HIV prevalence in 161 countries around the world ^[1]^. The country models are also applied to measure the performance of aniretroviral therapy (ART) and Prevention of Mother-To-Child Transmission (PMTCT) programs. These country models are developed and maintained by a team of country expert and can be accessed via request through UNIAIDS (https://www.unaids.org/en/dataanalysis/datatools/spectrum-epp). For the purpose of this study, we obtained formal approval to access and apply the 2019’s HIV SPECTRUM model in Kenya, and joined efforts with a team of experts at the Avenir Health to inform and oversee our analysis with this model.

Each SPECTRUM model consists of several simulation modules managing various aspects of population and disease dynamics. A detailed description of the methods and assumptions is available in the Spectrum/AIM manual (<http://www.avenirhealth.org/software-spectrum.php>). The national HIV SPECTRUM models consists of two main components including DemProj and AIDS impact model (AIM). DemProj represents the population by age and sex at a local- or national-level, and models population dynamics through fertility, mortality, and migration rates. AIM model projects the consequences of the HIV epidemic in terms of the number of people living with HIV and HIV-related deaths by age and sex. The Kenyan national AIM model is calibrated to the 2019 official HIV estimates from national AIDS control council. The final year of data entered into the file is as of December 2018.

## National Spectrum GOALS model

The Spectrum Goals model helps efforts to respond to HIV epidemic by estimating the effect of HIV interventions and resource allocation on HIV infections and deaths. Goals provides an extended model of HIV transmissions among different risk-groups (heterosexual, drug users, men who have sex with men and sex workers), and incorporate estimates of ART coverage to project ongoing transmissions. In collaboration with Avenir Health, we extended the Kenya’s Spectrum model to simulate HIV infections via the Goals module, and calibrated the underlying parameters to the official estimates of HIV incidence from 1970 to 2018.  The national Goals model was used to contrast two set of scenarios: 1) a baseline scenario representing fix levels of ART coverage at 2018’s value over time, and 2) an intervention scenario with improved ART coverage through the intervention period (2019-2033) and fixed levels of coverage over the following years. the main outcome of interest included the percent reduction in HIV incidence and HIV-related deaths.

## Regional SPECTRUM models

To further assess the impact of integration at a local level, we applied the Kenya’s regional Spectrum models in Nairobi, Coast and Central, calibrated to 2019’s official estimates. Given the difficulties in developing regional Goals models, and lack of data required to inform local models on size and contribution of various risk-groups to HIV incidence at each local setting, we adopted a simplifying assumption by modeling the projecting the impact of HIV interventions (in terms of reductions in HIV incidence) at a national level (via the Goals model) and applying the estimated annual reductions in HIV incidence to regional AIM models in order to project the future size of epidemic and HIV-related deaths at a local level.

## Modeling procedure for HIV scenarios via spectrum

At the national level, we created two separate Goals models, set up according to the baseline and intervention scenarios described previously—one with fixed ART coverage and one with improved ART coverage. We then pulled the HIV incidence trend from 2019 to 2033 in both scenarios and calculated the reduction in HIV incidence in the intervention scenario relative to the baseline scenario. Because the regional models are AIM files, they do not have the same ability as Goals files to estimate the impact of interventions (i.e., improved ART coverage) on HIV incidence. Thus, we implemented the regional baseline scenario by replicating the HIV incidence trend that was observed in the national baseline Goals model, and then created the intervention scenario by applying the same relative reduction observed in the national intervention model. This methodology allowed us to then generate the epidemic size and number of HIV-related deaths in each scenario at the regional level.

## Uncertainty analysis

Given the deterministic nature of Spectrum model, we applied additional uncertainty tools to generate uncertainty ranges around the main HIV-related outcomes presented in Table 4 in the main manuscript. Using the national Goals model, we generated 1,000 random simulations by permuting the epidemiological and behavioral parameters. The projected HIV prevalence in each simulation was compared against historical data, and simulations were weighted based on the goodness of fit. The weighted simulations were re-sampled 1,000 times, and the 95% uncertainty ranges around each outcome was calculated. Projections were made for both the baseline and intervention scenario models into the future. Due to unavailability of Goals models at a regional-level, we applied the same ratio of uncertainty ranges from the national model to all regional outputs.

# Assessing the epidemiological burden of hypertension and diabetes in Kenya

Kenya STEPwise survey for non-communicable diseases was the first nationwide cross-sectional survey among adults age 18-69 that providing comprehensive information on risk factors of the diseases. The sample size for STEPs survey was 6,000 individuals, and 4,500 individuals were eligible (no missing age and sex) for further estimates in the total sample. It was undertaken through the fifth National Sample Surveys and Evaluation Programme (NASSEP V) sampling frame. STEPwise survey used a three-stage cluster sample design which including behavioral risk factors (includes tobacco use, alcohol use, diet, exercises), physical measurements (includes blood pressure, heart rate, height, weight, waist and hip circumference) and biochemical measurements (includes blood glucose and cholesterol).

The regional prevalence of hypertension and diabetes mellitus in Kenya was estimated based on the Kenya STEPwise survey. Hypertension or raised blood pressure was defined as “systemic blood pressure ≥ 140 mmHg and/or diastolic blood pressure ≥ 90 mmHg or currently on medication for raised blood pressure”. Diabetes or raised blood glucose was defined as “plasma venous value ≥ 7.0 mmol/L or currently on medication for raised cholesterol”. (Kenya National Bureau of Statistics, 2015). Based on the EpiInfo programs (<https://www.who.int/ncds/surveillance/steps/resources/database/en/>) they used to analyze the results in the WHO report, we transferred and recoded these two main programs (MbloodpressureWT and BglucoseWT) into Stata and compared our outcomes to make sure that we used a correct weighting methods in our analysis. Then we created the variable of the region of Kenya according to the county names in the STEPwise dataset and used the same sample size and weights to extract the prevalence of hypertension and diabetes mellitus in the regional level. (Kenya region Wikipedia [https://en.wikipedia.org/wiki/Regions_of_Kenya](https://en.wikipedia.org/wiki/Provinces_of_Kenya)). Results were adjusted and weighted based on the survey sampling. All analysis was conducted in Stata 14.

## Regional heterogeneities in prevalence of hypertension and diabetes in Kenya

Using STEPwise dataset, we estimated the prevalence of hypertension and diabetes at a local and national level (Table 1). Figure 1 shows the heterogeneity in estimated burden of diseases at the county and region-level in Kenya. These heterogeneities may be driven by a combination of local factors relating to population size, age/sex decomposition, healthcare access, social norms, nutrition, etc.

Table 1: Estimated prevalence of hypertension and diabetes in Kenya a region- and national-level from the 2015 STEPwise survey. The prevalence included the prevalence of hypertension only, prevalence of diabetes only and prevalence of both hypertension and diabetes by sex and region.

| **Hypertension only** | | | | | | | | | | | | |
| --- | --- | --- | --- | --- | --- | --- | --- | --- | --- | --- | --- | --- |
|  | **Men** | | | | **Women** | | | | **Total** | | | |
| **Region** | **Prevalence** | **SE** | **95%CI LB** | **95%CI HB** | **Prevalence** | **SE** | **95%CI LB** | **95%CI HB** | **Prevalence** | **SE** | **95%CI LB** | **95%CI HB** |
| **Coast** | 0.226 | 0.049 | 0.144 | 0.337 | 0.159 | 0.032 | 0.106 | 0.232 | 0.197 | 0.035 | 0.137 | 0.274 |
| **North Eastern** | 0.231 | 0.074 | 0.116 | 0.406 | 0.121 | 0.02 | 0.086 | 0.166 | 0.156 | 0.033 | 0.102 | 0.232 |
| **Eastern** | 0.278 | 0.029 | 0.225 | 0.339 | 0.268 | 0.027 | 0.217 | 0.325 | 0.273 | 0.022 | 0.232 | 0.317 |
| **Central** | 0.34 | 0.049 | 0.251 | 0.443 | 0.346 | 0.041 | 0.27 | 0.43 | 0.343 | 0.03 | 0.287 | 0.404 |
| **Rift Valley** | 0.251 | 0.026 | 0.202 | 0.306 | 0.199 | 0.023 | 0.157 | 0.248 | 0.224 | 0.021 | 0.186 | 0.268 |
| **Western** | 0.267 | 0.041 | 0.195 | 0.355 | 0.225 | 0.041 | 0.154 | 0.315 | 0.247 | 0.023 | 0.205 | 0.294 |
| **Nyanza** | 0.204 | 0.041 | 0.135 | 0.296 | 0.2 | 0.019 | 0.165 | 0.239 | 0.202 | 0.027 | 0.154 | 0.259 |
| **Nairobi** | 0.148 | 0.044 | 0.081 | 0.256 | 0.058 | 0.018 | 0.031 | 0.105 | 0.105 | 0.026 | 0.065 | 0.167 |
| **Total** | 0.245 | 0.017 | 0.213 | 0.28 | 0.205 | 0.014 | 0.179 | 0.234 | 0.225 | 0.012 | 0.201 | 0.25 |
| **Diabetes only** | | | | | | | | | | | | |
|  | **Men** | | | | **Women** | | | | **Total** | | | |
| **Region** | **Prevalence** | **SE** | **95%CI LB** | **95%CI HB** | **Prevalence** | **SE** | **95%CI LB** | **95%CI HB** | **Prevalence** | **SE** | **95%CI LB** | **95%CI HB** |
| **Coast** | 0.031 | 0.015 | 0.012 | 0.08 | 0.004 | 0.004 | 0.001 | 0.023 | 0.019 | 0.009 | 0.008 | 0.047 |
| **North Eastern** | 0 | 0 | 0 | 0 | 0.031 | 0.012 | 0.014 | 0.066 | 0.021 | 0.009 | 0.009 | 0.047 |
| **Eastern** | 0.009 | 0.007 | 0.002 | 0.038 | 0.013 | 0.006 | 0.006 | 0.03 | 0.011 | 0.004 | 0.005 | 0.022 |
| **Central** | 0.005 | 0.005 | 0.001 | 0.037 | 0.007 | 0.005 | 0.002 | 0.03 | 0.006 | 0.004 | 0.002 | 0.02 |
| **Rift Valley** | 0.003 | 0.002 | 0.001 | 0.01 | 0.006 | 0.003 | 0.002 | 0.016 | 0.004 | 0.002 | 0.002 | 0.011 |
| **Western** | 0.011 | 0.007 | 0.003 | 0.036 | 0.01 | 0.009 | 0.002 | 0.053 | 0.011 | 0.005 | 0.004 | 0.027 |
| **Nyanza** | 0.006 | 0.004 | 0.001 | 0.024 | 0.005 | 0.003 | 0.001 | 0.018 | 0.005 | 0.003 | 0.002 | 0.015 |
| **Nairobi** | 0 | 0 | 0 | 0 | 0 | 0 | 0 | 0 | 0 | 0 | 0 | 0 |
| **Total** | 0.008 | 0.002 | 0.004 | 0.014 | 0.008 | 0.002 | 0.005 | 0.013 | 0.008 | 0.002 | 0.005 | 0.012 |
| **Hypertension and Diabetes** | | | | | | | | | | | | |
|  | **Men** | | | | **Women** | | | | **Total** | | | |
| **Region** | **Prevalence** | **SE** | **95%CI LB** | **95%CI HB** | **Prevalence** | **SE** | **95%CI LB** | **95%CI HB** | **Prevalence** | **SE** | **95%CI LB** | **95%CI HB** |
| **Coast** | 0 | 0 | 0 | 0 | 0.003 | 0.002 | 0.001 | 0.012 | 0.001 | 0.001 | 0 | 0.005 |
| **North Eastern** | 0 | 0 | 0 | 0 | 0.007 | 0.005 | 0.002 | 0.026 | 0.004 | 0.003 | 0.001 | 0.017 |
| **Eastern** | 0.009 | 0.008 | 0.002 | 0.05 | 0.022 | 0.009 | 0.01 | 0.048 | 0.016 | 0.008 | 0.006 | 0.043 |
| **Central** | 0.004 | 0.005 | 0.001 | 0.032 | 0.034 | 0.012 | 0.016 | 0.068 | 0.019 | 0.007 | 0.009 | 0.04 |
| **Rift Valley** | 0.004 | 0.003 | 0.001 | 0.014 | 0.004 | 0.002 | 0.002 | 0.009 | 0.004 | 0.002 | 0.001 | 0.01 |
| **Western** | 0.012 | 0.007 | 0.004 | 0.039 | 0.003 | 0.003 | 0.001 | 0.017 | 0.008 | 0.004 | 0.003 | 0.022 |
| **Nyanza** | 0.001 | 0.001 | 0 | 0.008 | 0.007 | 0.004 | 0.002 | 0.023 | 0.004 | 0.002 | 0.001 | 0.013 |
| **Nairobi** | 0.024 | 0.022 | 0.004 | 0.133 | 0.042 | 0.012 | 0.024 | 0.074 | 0.033 | 0.017 | 0.012 | 0.087 |
| **Total** | 0.007 | 0.003 | 0.003 | 0.019 | 0.015 | 0.003 | 0.009 | 0.024 | 0.011 | 0.003 | 0.007 | 0.019 |


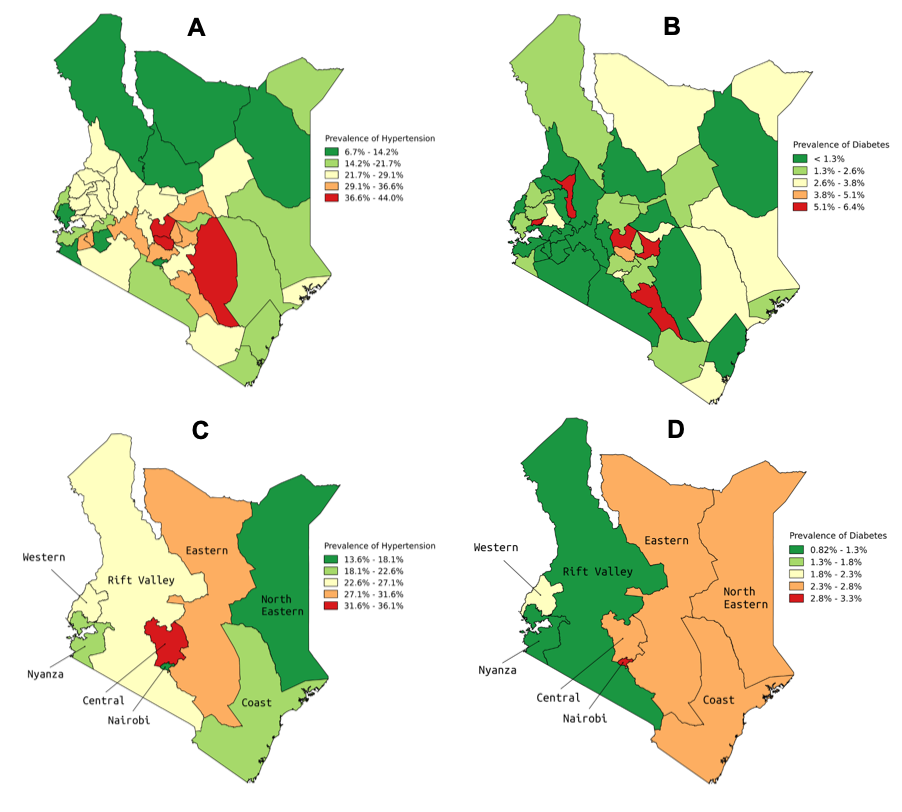


Figure 1: heterogeneities in prevalence of hypertension and diabetes at the county- and region-level in Kenya. Panel A and B showed the prevalence of hypertension (A) and prevalence of diabetes (B) at the county level. Panel C and D showed the prevalence of hypertension (C) and prevalence of diabetes (D) at the region level.

## Selecting representative regions:

In order to assess the impact local heterogeneities in terms of population demographics, NCD burden and HIV epidemic, on potential impact of integrating services for HIV/NCDs in Kenya, we extend our analysis to include representative regions in Kenya. Figure 2 provide a schematic comparison of HIV and NCD burden at a region-level. The values suggested wide heterogeneities in hypertension prevalence, ranging from 11% in Nairobi to 35% in the Central region. Diabetes prevalence was less heterogenous, ranging from 0.8% in Rift Valley to 3.28% in Nairobi. With an incidence of 3.2 per 1000 person year, Nyanza had the highest HIV incidence rate, followed by Nairobi with an incidence of 1.2. ART coverage ranged from 41% in North Eastern to 83% in Western. Consequently, we chose 3 regions including Central (representative of a high NCD burden), Nairobi (representative of a high HIV burden) and Coast (representative of a moderate HIV/NCD burden) region.


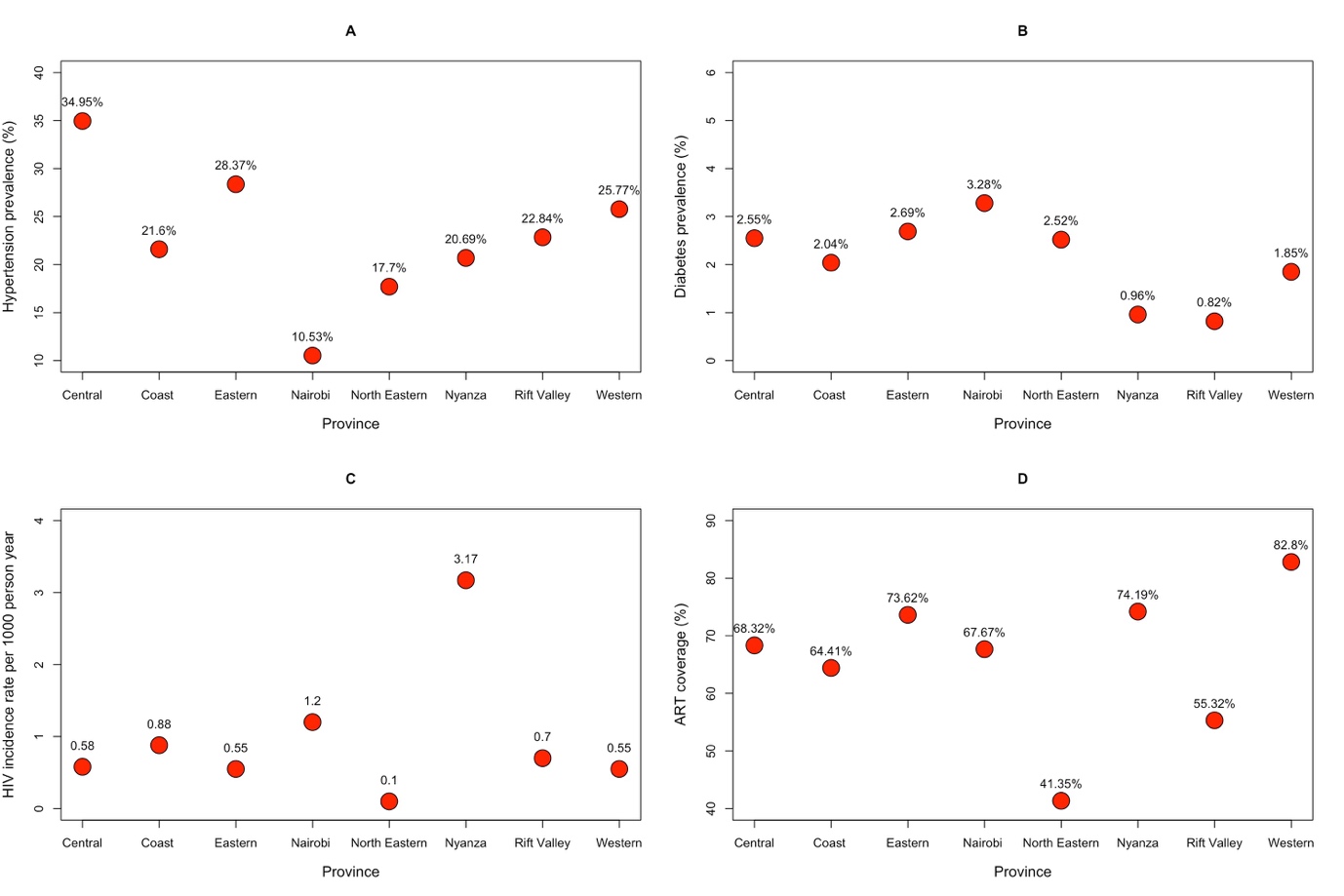


Figure 2: Regional heterogeneities in HIV and NCD in Kenya. The X-axis present the regions in Kenya. Shown in panel A and B are the prevalence of hypertension and diabetes at a region level, estimated from the 2015 STEPwise survey. Panel C and D show the incidence of HIV and ART coverage estimated from the Spectrum model.

# CVD microsimulation model

## Model structure

The underlying structure of our individual based CVD model is based on a recently published study by Subramanian et al. (2019)^[2]^. Each individual in our model is described by age, sex, HIV status, ART usage, and underlying CVD risk category. The CVD risk categories are stratified based on 10-year risk of CVD events, hypertension (blood pressure greater or less than 140/90 mmHg) and diabetes (plasma venous value ≥ 7.0 mmol/L or currently on medication for diabetes). To estimate the proportion of Kenyan population falling into each risk category, we applied data from the 2015 STEPwise survey in Kenya^[3]^. Using reported measures of blood pressure, fasting blood glucose, cholesterol and HDL, as well as, information on age, sex and tobacco use, we first estimated the 10-year risk of a CVD event for each individuals through the Framingham model^[4]^. We further used these risk scores along with individual-level measures of blood pressure (systolic blood pressure-SBP & diastolic blood pressure-DBP) and diabetes status to assign individuals into 8 risk categories as described in Table 1 in the main manuscript. As individuals age with time, the model allows for transitions to higher-risk categories (Figure 3) and also for reduction in that risk through treatment.

Finally, we estimated the population proportion falling within each risk category by sex and 5-year age category (see below). We applied these risk categories as a composite measure of risk for future CVD events including potential cardiovascular heart disease (CHD) (e.g., cardiac arrest, myocardial infraction [MI], or angina) and stroke, and estimated the annual risk of a first CHD and stroke event for each risk category from the corresponding Framingham risk profiles ^[5, 6]^.


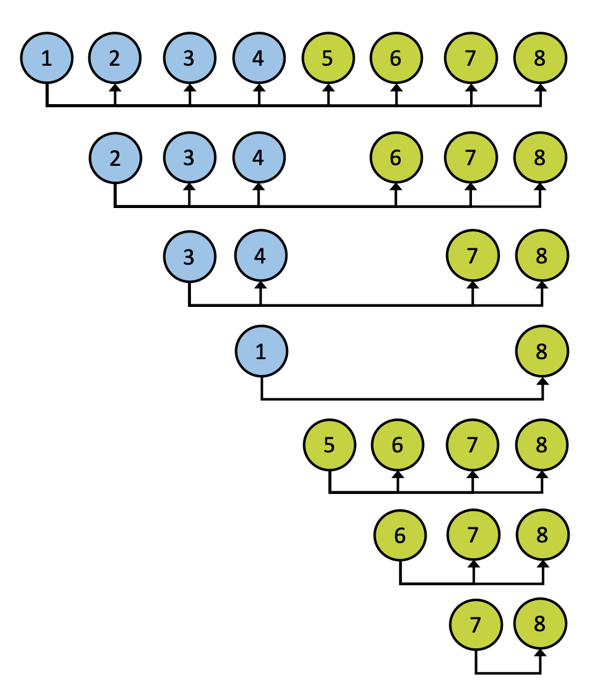


Figure 3: Possible transitions between risk categories in the HIV/CVD microsimulation. As individuals age, they can move to higher risk categories over time. We assume that diabetes is an absorbing state, and while non-diabetic individuals (risk category 5 to 8, shaded in blue) can develop diabetes over time, those with diabetes (risk category 5 to 8, shaded in pink) can never return to non-diabetic risk categories.

## Developing population “risk profile”

In order to quantify the epidemiological and economic burden of hypertension and diabetes, we focused on the subsequent effect of these diseases on incidence of potential CVD events that could result in death or disability. For this purpose, we defined eight risk categories based on binary classifications of Framingham-calculated 10-year CVD risk (greater or less than 10%), hypertension (blood pressure greater or less than 140/90 mmHg) and diabetes status (plasma venous value ≥ 7.0 mmol/L or currently on medication for diabetes) and estimated the proportion of population that fell within each risk category by sex- and 5-year age-strata at a national level (Table 2).

Given the existing differences between the Kenyan population and STEP survey in terms of age- and sex- decomposition, we further adjusted the national risk profile to match the reported prevalence of hypertension and diabetes (among those 15-70 years old) at a national level. For this purpose, we used the estimated population distribution by age/sex in 2015 from the Spectrum model and defined three adjustment coefficients to tune the proportion of population with hypertension only, diabetes only, and those with hypertension and diabetes. Using this approach, we derived the adjusted risk profile for the population of Kenya in 2015 as shown in Table 3. For individuals falling outside these age categories, we assume that those younger than 15 years fall in risk category 1 (no diabetes or hypertension and low CVD risk), and those older than 70 years old are similar to the last age-group (65-70 years).

Table 2: Unadjusted population risk profile at a national level. Values represent the proportion of population falling into each risk category by age group and sex using data from the 2015 STEPwise survey. Cells with no observation show a value of 0. Each panel is highlighted based on cells’ values ranging from lowest (blue) to highest (pink), suggesting an increasing trend in risk category as individual age.

|  | **Risk category** | | | | | | | |
| --- | --- | --- | --- | --- | --- | --- | --- | --- |
|  | 1 | 2 | 3 | 4 | 5 | 6 | 7 | 8 |
| **Male** | | | | | | | | |
| Ages: [15 , 20) | 0.82667 | 0 | 0.17333 | 0 | 0 | 0 | 0 | 0 |
| [20 , 25) | 0.84804 | 0 | 0.14706 | 0 | 0.0049 | 0 | 0 | 0 |
| [25 , 30) | 0.77027 | 0 | 0.21171 | 0 | 0.01351 | 0 | 0.0045 | 0 |
| [30 , 35) | 0.79574 | 0 | 0.18723 | 0 | 0.01277 | 0 | 0.00426 | 0 |
| [35 , 40) | 0.7243 | 0 | 0.24766 | 0.01402 | 0.01402 | 0 | 0 | 0 |
| [40 , 45) | 0.67033 | 0.03297 | 0.24725 | 0.02747 | 0.01099 | 0 | 0.00549 | 0.00549 |
| [45 , 50) | 0.5748 | 0.04724 | 0.11811 | 0.20472 | 0.02362 | 0.00787 | 0 | 0.02362 |
| [50 , 55) | 0.5 | 0.14286 | 0.15306 | 0.15306 | 0 | 0 | 0 | 0.05102 |
| [55 , 60) | 0.34043 | 0.21277 | 0.05319 | 0.31915 | 0 | 0.03191 | 0 | 0.04255 |
| [60 , 65) | 0.25287 | 0.26437 | 0 | 0.42529 | 0 | 0.03448 | 0 | 0.02299 |
| [65 , 70) | 0.08621 | 0.32759 | 0.01724 | 0.5 | 0 | 0 | 0 | 0.06897 |
| **Female** | | | | | | | | |
| Ages: [15 , 20) | 0.89516 | 0 | 0.09677 | 0 | 0.00806 | 0 | 0 | 0 |
| [20 , 25) | 0.91667 | 0 | 0.08333 | 0 | 0 | 0 | 0 | 0 |
| [25 , 30) | 0.8557 | 0 | 0.12658 | 0 | 0.01266 | 0 | 0.00506 | 0 |
| [30 , 35) | 0.85754 | 0 | 0.12849 | 0 | 0.01397 | 0 | 0 | 0 |
| [35 , 40) | 0.74434 | 0 | 0.2233 | 0.00324 | 0.02265 | 0 | 0.00647 | 0 |
| [40 , 45) | 0.71064 | 0 | 0.22553 | 0.01277 | 0.02128 | 0 | 0.01702 | 0.01277 |
| [45 , 50) | 0.58286 | 0 | 0.34286 | 0.02286 | 0.02857 | 0 | 0.01714 | 0.00571 |
| [50 , 55) | 0.49032 | 0 | 0.35484 | 0.05161 | 0.0129 | 0.00645 | 0.00645 | 0.07742 |
| [55 , 60) | 0.48905 | 0.0073 | 0.27737 | 0.14599 | 0.0073 | 0.0073 | 0 | 0.06569 |
| [60 , 65) | 0.36885 | 0.04098 | 0.19672 | 0.31148 | 0.0082 | 0.02459 | 0.0082 | 0.04098 |
| [65 , 70) | 0.29808 | 0.02885 | 0.13462 | 0.40385 | 0 | 0.01923 | 0 | 0.11538 |

Table 3: Adjusted population risk profile at a national level. Values represent the proportion of population falling into each risk category by age group and sex after adjustment for the Kenyan population demographics in year 2015. Cells with no observation show a value of 0. Each panel is highlighted based on cells’ values ranging from lowest (blue) to highest (pink), suggesting an increasing trend in risk category as individual age.

| **Risk category** | | | | | | | | |
| --- | --- | --- | --- | --- | --- | --- | --- | --- |
| **Male** | **1** | **2** | **3** | **4** | **5** | **6** | **7** | **8** |
| [15 , 20) | 0.8145 | 0 | 0.1855 | 0 | 0 | 0 | 0 | 0 |
| [20 , 25) | 0.838 | 0 | 0.1574 | 0 | 0.0047 | 0 | 0 | 0 |
| [25 , 30) | 0.7561 | 0 | 0.2263 | 0 | 0.0126 | 0 | 0.005 | 0 |
| [30 , 35) | 0.7833 | 0 | 0.2001 | 0 | 0.0119 | 0 | 0.0047 | 0 |
| [35 , 40) | 0.7063 | 0.001 | 0.2645 | 0.015 | 0.0133 | 0 | 0 | 0 |
| [40 , 45) | 0.6527 | 0.031 | 0.2639 | 0.0291 | 0.0101 | 0.001 | 0.0061 | 0.0061 |
| [45 , 50) | 0.5677 | 0.0333 | 0.1264 | 0.2178 | 0.0224 | 0.0062 | 0 | 0.0262 |
| [50 , 55) | 0.4874 | 0.1316 | 0.1632 | 0.1604 | 0 | 0.001 | 0 | 0.0564 |
| [55 , 60) | 0.3367 | 0.192 | 0.0569 | 0.3392 | 0 | 0.028 | 0 | 0.0472 |
| [60 , 65) | 0.2529 | 0.2363 | 0 | 0.4538 | 0 | 0.0315 | 0 | 0.0255 |
| [65 , 70) | 0.0846 | 0.2912 | 0.0184 | 0.5287 | 0 | 0.001 | 0 | 0.0762 |
| **Female** |  |  |  |  |  |  |  |  |
| [15 , 20) | 0.8941 | 0 | 0.1016 | 0 | 0.0043 | 0 | 0 | 0 |
| [20 , 25) | 0.9125 | 0 | 0.0875 | 0 | 0 | 0 | 0 | 0 |
| [25 , 30) | 0.8553 | 0 | 0.1338 | 0 | 0.0076 | 0 | 0.0033 | 0 |
| [30 , 35) | 0.8577 | 0 | 0.1349 | 0 | 0.0074 | 0 | 0 | 0 |
| [35 , 40) | 0.743 | 0.001 | 0.2353 | 0.0034 | 0.0131 | 0 | 0.0042 | 0 |
| [40 , 45) | 0.7082 | 0.001 | 0.2394 | 0.0156 | 0.0142 | 0.0022 | 0.0111 | 0.0083 |
| [45 , 50) | 0.5779 | 0.001 | 0.3622 | 0.025 | 0.0181 | 0.001 | 0.0111 | 0.0037 |
| [50 , 55) | 0.4787 | 0.0005 | 0.3737 | 0.0677 | 0.008 | 0.017 | 0.0042 | 0.0503 |
| [55 , 60) | 0.4786 | 0.0034 | 0.2912 | 0.1648 | 0.0039 | 0.0154 | 0 | 0.0427 |
| [60 , 65) | 0.3629 | 0.037 | 0.208 | 0.3342 | 0.0058 | 0.0202 | 0.0053 | 0.0266 |
| [65 , 70) | 0.2914 | 0.0177 | 0.1414 | 0.4442 | 0 | 0.0304 | 0 | 0.075 |

## Estimating population risk profile at each region

Using the estimated population distribution by age/sex in 2015 from the regional Spectrum models, we applied a similar adjustment approach to adjust the national risk profile for each region. Our goal was to match the estimated prevalence of hypertension and diabetes at the regional level (as shown in table 3 of the manuscript) in year 2015. Table 4-6 show the estimated risk profiles for Nairobi, Coast and Central regions.

Table 4: Adjusted population risk profile for Nairobi. Values represent the proportion of population falling into each risk category by age group and sex after adjustment for Nairobi’s population demographics in 2015. Cells with no observation show a value of 0. Each panel is highlighted based on cells’ values ranging from lowest (blue) to highest (pink).

| **Risk category** | | | | | | | | | |
| --- | --- | --- | --- | --- | --- | --- | --- | --- | --- |
| **Male** | **1** | **2** | **3** | **4** | **5** | **6** | **7** | **8** |  |
| [15 , 20) | 0.883 | 0 | 0.117 | 0 | 0 | 0 | 0 | 0 |  |
| [20 , 25) | 0.9007 | 0 | 0.0993 | 0 | 0 | 0 | 0 | 0 |  |
| [25 , 30) | 0.8481 | 0 | 0.1379 | 0 | 0.001 | 0 | 0.013 | 0 |  |
| [30 , 35) | 0.865 | 0 | 0.1217 | 0 | 0.001 | 0 | 0.0123 | 0 |  |
| [35 , 40) | 0.8188 | 0.0046 | 0.1672 | 0.0095 | 0 | 0 | 0 | 0 |  |
| [40 , 45) | 0.7523 | 0.0414 | 0.1597 | 0.0132 | 0.001 | 0.001 | 0.0157 | 0.0157 |  |
| [45 , 50) | 0.6222 | 0.1189 | 0.0779 | 0.1131 | 0 | 0.001 | 0 | 0.0669 |  |
| [50 , 55) | 0.5238 | 0.1835 | 0.0985 | 0.0523 | 0 | 0.001 | 0 | 0.141 |  |
| [55 , 60) | 0.3435 | 0.3346 | 0.0345 | 0.168 | 0 | 0.001 | 0 | 0.1185 |  |
| [60 , 65) | 0.2472 | 0.4273 | 0 | 0.2593 | 0 | 0.001 | 0 | 0.0652 |  |
| [65 , 70) | 0.0861 | 0.4595 | 0.0109 | 0.255 | 0 | 0.0009 | 0 | 0.1875 |  |
| **Female** |  |  |  |  |  |  |  |  |  |
| [15 , 20) | 0.9574 | 0 | 0.0426 | 0 | 0 | 0 | 0 | 0 |  |
| [20 , 25) | 0.9633 | 0 | 0.0367 | 0 | 0 | 0 | 0 | 0 |  |
| [25 , 30) | 0.9294 | 0 | 0.0456 | 0 | 0.001 | 0 | 0.0241 | 0 |  |
| [30 , 35) | 0.9435 | 0 | 0.0565 | 0 | 0 | 0 | 0 | 0 |  |
| [35 , 40) | 0.8803 | 0.0018 | 0.0848 | 0.0014 | 0.001 | 0 | 0.0307 | 0 |  |
| [40 , 45) | 0.7959 | 0.0066 | 0.062 | 0.0009 | 0.0009 | 0.0009 | 0.0758 | 0.0568 |  |
| [45 , 50) | 0.7672 | 0.0122 | 0.113 | 0.001 | 0.001 | 0.001 | 0.0786 | 0.0262 |  |
| [50 , 55) | 0.5455 | 0.0275 | 0.1118 | 0.0008 | 0.0008 | 0.0008 | 0.0241 | 0.2888 |  |
| [55 , 60) | 0.5488 | 0.0811 | 0.1028 | 0.0008 | 0 | 0.0008 | 0 | 0.2656 |  |
| [60 , 65) | 0.4448 | 0.2191 | 0.0648 | 0.054 | 0.0009 | 0.0009 | 0.0359 | 0.1796 |  |
| [65 , 70) | 0.2958 | 0.2172 | 0.0469 | 0.0008 | 0 | 0.0008 | 0 | 0.4386 |  |

Table 5: Adjusted population risk profile for Central region. Values represent the proportion of population falling into each risk category by age group and sex after adjustment for Central region’s population demographics in 2015. Cells with no observation show a value of 0. Each panel is highlighted based on cells’ values ranging from lowest (blue) to highest (pink).

| **Risk category** | | | | | | | | |
| --- | --- | --- | --- | --- | --- | --- | --- | --- |
| **Male** | **1** | **2** | **3** | **4** | **5** | **6** | **7** | **8** |
| [15 , 20) | 0.7556 | 0 | 0.2444 | 0 | 0 | 0 | 0 | 0 |
| [20 , 25) | 0.79206 | 0 | 0.20735 | 0 | 0.00059 | 0 | 0 | 0 |
| [25 , 30) | 0.69536 | 0 | 0.29993 | 0 | 0.00304 | 0 | 0.00167 | 0 |
| [30 , 35) | 0.73021 | 0 | 0.26534 | 0 | 0.00287 | 0 | 0.00157 | 0 |
| [35 , 40) | 0.63084 | 0.00099 | 0.34687 | 0.01963 | 0.00167 | 0 | 0 | 0 |
| [40 , 45) | 0.57863 | 0.0217 | 0.35036 | 0.04047 | 0.00305 | 0.00173 | 0.00203 | 0.00203 |
| [45 , 50) | 0.53083 | 0.00097 | 0.16157 | 0.28727 | 0.00275 | 0.00814 | 0 | 0.00848 |
| [50 , 55) | 0.43724 | 0.0801 | 0.21582 | 0.23189 | 0 | 0.01607 | 0 | 0.01888 |
| [55 , 60) | 0.31862 | 0.11 | 0.075 | 0.4634 | 0 | 0.01723 | 0 | 0.01574 |
| [60 , 65) | 0.25287 | 0.12034 | 0 | 0.6069 | 0 | 0.01138 | 0 | 0.00851 |
| [65 , 70) | 0.07914 | 0.12259 | 0.02431 | 0.72672 | 0 | 0.02172 | 0 | 0.02552 |
| **Female** |  |  |  |  |  |  |  |  |
| [15 , 20) | 0.82565 | 0 | 0.16839 | 0 | 0.00597 | 0 | 0 | 0 |
| [20 , 25) | 0.855 | 0 | 0.145 | 0 | 0 | 0 | 0 | 0 |
| [25 , 30) | 0.76532 | 0 | 0.21797 | 0 | 0.00709 | 0 | 0.00962 | 0 |
| [30 , 35) | 0.76609 | 0 | 0.22358 | 0 | 0.01034 | 0 | 0 | 0 |
| [35 , 40) | 0.583 | 0.001 | 0.38433 | 0.00561 | 0.0138 | 0 | 0.01226 | 0 |
| [40 , 45) | 0.53999 | 0.00098 | 0.37826 | 0.01619 | 0.00795 | 0.00098 | 0.03179 | 0.02385 |
| [45 , 50) | 0.32949 | 0.00098 | 0.57647 | 0.03642 | 0.01315 | 0.00098 | 0.03189 | 0.01063 |
| [50 , 55) | 0.21627 | 0.00094 | 0.57508 | 0.05144 | 0.00622 | 0.00094 | 0.01147 | 0.13766 |
| [55 , 60) | 0.25395 | 0.00089 | 0.42901 | 0.19951 | 0.0048 | 0.00089 | 0 | 0.11095 |
| [60 , 65) | 0.19016 | 0.00084 | 0.28566 | 0.44166 | 0.00201 | 0.00084 | 0.01314 | 0.06569 |
| [65 , 70) | 0.15211 | 0.00077 | 0.17953 | 0.49879 | 0 | 0.00077 | 0 | 0.16803 |

Table 6: Adjusted population risk profile for Coast region. Values represent the proportion of population falling into each risk category by age group and sex after adjustment for Coast region’s population demographics in 2015. Cells with no observation show a value of 0. Each panel is highlighted based on cells’ values ranging from lowest (blue) to highest (pink).

| **Risk category** | | | | | | | | |
| --- | --- | --- | --- | --- | --- | --- | --- | --- |
| **Male** | **1** | **2** | **3** | **4** | **5** | **6** | **7** | **8** |
| [15 , 20) | 0.83533 | 0 | 0.16467 | 0 | 0 | 0 | 0 | 0 |
| [20 , 25) | 0.84828 | 0 | 0.13971 | 0 | 0.01201 | 0 | 0 | 0 |
| [25 , 30) | 0.76126 | 0 | 0.20338 | 0 | 0.03536 | 0 | 0 | 0 |
| [30 , 35) | 0.7866 | 0 | 0.18 | 0 | 0.0334 | 0 | 0 | 0 |
| [35 , 40) | 0.71636 | 0.0007 | 0.23528 | 0.01332 | 0.03435 | 0 | 0 | 0 |
| [40 , 45) | 0.66676 | 0.03434 | 0.23764 | 0.02885 | 0.02967 | 0.00275 | 0 | 0 |
| [45 , 50) | 0.54646 | 0.04606 | 0.1122 | 0.2063 | 0.05787 | 0.0311 | 0 | 0 |
| [50 , 55) | 0.50765 | 0.15051 | 0.14541 | 0.17092 | 0 | 0.02551 | 0 | 0 |
| [55 , 60) | 0.34309 | 0.18245 | 0.05053 | 0.32447 | 0 | 0.09947 | 0 | 0 |
| [60 , 65) | 0.25287 | 0.23563 | 0 | 0.41552 | 0 | 0.09598 | 0 | 0 |
| [65 , 70) | 0.08707 | 0.35259 | 0.01638 | 0.50948 | 0 | 0.03448 | 0 | 0 |
| **Female** |  |  |  |  |  |  |  |  |
| [15 , 20) | 0.9221 | 0 | 0.07691 | 0 | 0.00099 | 0 | 0 | 0 |
| [20 , 25) | 0.93333 | 0 | 0.06667 | 0 | 0 | 0 | 0 | 0 |
| [25 , 30) | 0.89558 | 0 | 0.10257 | 0 | 0.00099 | 0 | 0.00085 | 0 |
| [30 , 35) | 0.89731 | 0 | 0.1017 | 0 | 0.00099 | 0 | 0 | 0 |
| [35 , 40) | 0.81594 | 0.00064 | 0.17879 | 0.00255 | 0.00099 | 0 | 0.00108 | 0 |
| [40 , 45) | 0.78498 | 0.00253 | 0.18585 | 0.01537 | 0.00099 | 0.00525 | 0.00287 | 0.00215 |
| [45 , 50) | 0.69041 | 0.00451 | 0.27755 | 0.02037 | 0.00099 | 0.00234 | 0.00287 | 0.00096 |
| [50 , 55) | 0.57897 | 0.02113 | 0.28446 | 0.07288 | 0.00099 | 0.02741 | 0.00109 | 0.01307 |
| [55 , 60) | 0.55355 | 0.04861 | 0.22055 | 0.14318 | 0.00099 | 0.02202 | 0 | 0.0111 |
| [60 , 65) | 0.42022 | 0.14443 | 0.16005 | 0.26499 | 0.001 | 0.001 | 0.00139 | 0.00694 |
| [65 , 70) | 0.325 | 0.14231 | 0.10769 | 0.37096 | 0 | 0.03442 | 0 | 0.01962 |

## Estimating the risk of first CHD and stroke events

The risk of first CHD and stroke events for each age-, sex-, and risk strata was derived from corresponding Framingham calculators ^[5, 6]^. To this end, we estimated the risk of CHD and stroke for all individuals in the sample, and reported the median risks for individuals falling within each age/sex/risk strata. Given the lack of observation for some combination of age, sex and risk, we further adjusted the missing values in Table 7-8 by taking the average difference between the cells immediately to the left and above such that the values are increasing from left-to-right (from lower to higher risk categories) and top-to-bottom (from younger to older ages). Final values are summarized in Table 9-10. For individuals falling outside these age categories, we set the risks to those of the closest age-groups. We apply these risks in all CVD models at a national and regional levels.

Table 7: Unadjusted estimates of annual risks for Stroke by risk category, sex and age for total population of Kenya. Each cell represent the average value of annual risk for the corresponding CVD event among all individuals falling with that strata. Cells with no observation are marked as “NA”. Each panel is highlighted based on cells’ values ranging from lowest (blue) to highest (pink).

|  | **Risk category** | | | | | | | | | | | | |  |
| --- | --- | --- | --- | --- | --- | --- | --- | --- | --- | --- | --- | --- | --- | --- |
| **Male** | **1** | **2** | **3** | **4** | | **5** | | **6** | | **7** | | **8** | |  |
| Ages: [15 , 20) | 0.0031 | NA | 0.0046 | NA | | NA | | NA | | NA | | NA | |  |
| [20 , 25) | 0.0035 | NA | 0.0046 | NA | | 0.0051 | | NA | | NA | | NA | |  |
| [25 , 30) | 0.0034 | NA | 0.0052 | NA | | 0.0044 | | NA | | 0.0051 | | NA | |  |
| [30 , 35) | 0.0036 | NA | 0.0049 | NA | | 0.0048 | | NA | | 0.0072 | | NA | |  |
| [35 , 40) | 0.0035 | NA | 0.0051 | 0.0065 | | 0.0044 | | NA | | NA | | NA | |  |
| [40 , 45) | 0.0036 | 0.0049 | 0.0049 | 0.0077 | | 0.0036 | | NA | | 0.0062 | | 0.0116 | |  |
| [45 , 50) | 0.0036 | 0.0046 | 0.0044 | 0.0068 | | 0.0044 | | 0.0051 | | NA | | 0.008 | |  |
| [50 , 55) | 0.0032 | 0.0048 | 0.0055 | 0.0054 | | NA | | NA | | NA | | 0.0058 | |  |
| [55 , 60) | 0.0038 | 0.0049 | 0.0051 | 0.0066 | | NA | | 0.0044 | | NA | | 0.0131 | |  |
| [60 , 65) | 0.0046 | 0.0054 | NA | 0.009 | | NA | | 0.009 | | NA | | 0.0078 | |  |
| [65 , 70) | 0.0049 | 0.0061 | 0.0083 | 0.0116 | | NA | | NA | | NA | | 0.0151 | |  |
| **Female** | | | | |  | |  | |  | |  | |  | |
| Ages: [15 , 20) | 0.0013 | NA | 0.0021 | NA | | 0.002 | | NA | | NA | | NA | |  |
| [20 , 25) | 0.0013 | NA | 0.0023 | NA | | NA | | NA | | NA | | NA | |  |
| [25 , 30) | 0.0013 | NA | 0.0023 | NA | | 0.0028 | | NA | | 0.0036 | | NA | |  |
| [30 , 35) | 0.0014 | NA | 0.0022 | NA | | 0.002 | | NA | | NA | | NA | |  |
| [35 , 40) | 0.0014 | NA | 0.0023 | 0.0051 | | 0.0026 | | NA | | 0.0046 | | NA | |  |
| [40 , 45) | 0.0014 | NA | 0.0024 | 0.0051 | | 0.0033 | | NA | | 0.003 | | 0.0048 | |  |
| [45 , 50) | 0.0015 | NA | 0.0026 | 0.0062 | | 0.0037 | | NA | | 0.0037 | | 0.0094 | |  |
| [50 , 55) | 0.0015 | NA | 0.0025 | 0.0041 | | 0.002 | | 0.003 | | 0.0041 | | 0.0063 | |  |
| [55 , 60) | 0.0016 | 0.003 | 0.0027 | 0.0039 | | 0.003 | | 0.0062 | | NA | | 0.0075 | |  |
| [60 , 65) | 0.0023 | 0.0028 | 0.0042 | 0.0059 | | 0.0041 | | 0.0044 | | 0.0051 | | 0.0093 | |  |
| [65 , 70) | 0.0037 | 0.0041 | 0.005 | 0.0079 | | NA | | 0.0073 | | NA | | 0.0146 | |  |

Table 8: Unadjusted estimates of annual risks for CHD by risk category, sex and age for total population of Kenya. Each cell represent the average value of annual risk for the corresponding CVD event among all individuals falling with that strata. Cells with no observation are marked as “NA”. Each panel is highlighted based on cells’ values ranging from lowest (blue) to highest (pink).

|  | **Risk category** | | | | | | | | | | | | |  |
| --- | --- | --- | --- | --- | --- | --- | --- | --- | --- | --- | --- | --- | --- | --- |
| **Male** | **1** | **2** | **3** | **4** | | **5** | | **6** | | **7** | | **8** | |  |
| Ages: [15 , 20) | 0.001 | NA | 0.001 | NA | | NA | | NA | | NA | | NA | |  |
| [20 , 25) | 0.001 | NA | 0.001 | NA | | 0.001 | | NA | | NA | | NA | |  |
| [25 , 30) | 0.001 | NA | 0.0011 | NA | | 0.001 | | NA | | 0.001 | | NA | |  |
| [30 , 35) | 0.001 | NA | 0.001 | NA | | 0.001 | | NA | | 0.002 | | NA | |  |
| [35 , 40) | 0.0014 | NA | 0.0014 | 0.0085 | | 0.001 | | NA | | NA | | NA | |  |
| [40 , 45) | 0.0013 | 0.006 | 0.0015 | 0.0026 | | 0.001 | | NA | | 0.001 | | 0.003 | |  |
| [45 , 50) | 0.0024 | 0.0071 | 0.0024 | 0.006 | | 0.0013 | | 0.0105 | | NA | | 0.0048 | |  |
| [50 , 55) | 0.003 | 0.0066 | 0.0033 | 0.0076 | | NA | | NA | | NA | | 0.0047 | |  |
| [55 , 60) | 0.0044 | 0.0106 | 0.0043 | 0.0084 | | NA | | 0.0083 | | NA | | 0.0111 | |  |
| [60 , 65) | 0.006 | 0.0094 | NA | 0.0105 | | NA | | 0.0135 | | NA | | 0.0116 | |  |
| [65 , 70) | 0.0073 | 0.0106 | 0.0083 | 0.0137 | | NA | | NA | | NA | | 0.0122 | |  |
| **Female** | | | | |  | |  | |  | |  | |  | |
| Ages: [15 , 20) | 0 | NA | 0 | NA | | 0 | | NA | | NA | | NA | |  |
| [20 , 25) | 0 | NA | 0 | NA | | NA | | NA | | NA | | NA | |  |
| [25 , 30) | 0 | NA | 0 | NA | | 0 | | NA | | 0 | | NA | |  |
| [30 , 35) | 0 | NA | 0 | NA | | 0 | | NA | | NA | | NA | |  |
| [35 , 40) | 1E-05 | NA | 6E-05 | 0.001 | | 0 | | NA | | 0 | | NA | |  |
| [40 , 45) | 1E-05 | NA | 0.0002 | 0.0007 | | 0 | | NA | | 0 | | 0.0007 | |  |
| [45 , 50) | 0.0002 | NA | 0.0006 | 0.0013 | | 0 | | NA | | 0.0003 | | 0.002 | |  |
| [50 , 55) | 0.0005 | NA | 0.0012 | 0.0032 | | 0 | | 0.002 | | 0.001 | | 0.0021 | |  |
| [55 , 60) | 0.0012 | 0.003 | 0.0016 | 0.0041 | | 0.001 | | 0.0062 | | NA | | 0.004 | |  |
| [60 , 65) | 0.0014 | 0.0041 | 0.0022 | 0.0043 | | 0.001 | | 0.0024 | | 0.002 | | 0.0035 | |  |
| [65 , 70) | 0.0026 | 0.0044 | 0.003 | 0.0059 | | NA | | 0.0031 | | NA | | 0.0082 | |  |

Table 9: Adjusted estimates of annual risks for Stroke by risk category, sex and age for the total population of Kenya. Each cell represent the average value of annual risk for the corresponding CVD event among all individuals falling with that strata. missing observations are adjusted by taking the average difference between the cells immediately to the left and above such that the values are increasing from left-to-right (for higher risk categories) and top-to-bottom (for older ages). Each panel is highlighted based on cells’ values ranging from lowest (blue) to highest (pink).

|  | **Risk category** | | | | | | | |
| --- | --- | --- | --- | --- | --- | --- | --- | --- |
| **Male** | **1** | **2** | **3** | **4** | **5** | **6** | **7** | **8** |
| Ages: [15 , 20) | 0.0031 | 0.0031 | 0.0046 | 0.0046 | 0.0031 | 0.0031 | 0.0031 | 0.0031 |
| [20 , 25) | 0.0035 | 0.0035 | 0.0046 | 0.0046 | 0.0051 | 0.0051 | 0.0051 | 0.0051 |
| [25 , 30) | 0.0034 | 0.0034 | 0.0052 | 0.0052 | 0.0044 | 0.0044 | 0.0051 | 0.0051 |
| [30 , 35) | 0.0036 | 0.0036 | 0.0049 | 0.0049 | 0.0048 | 0.0048 | 0.0072 | 0.0072 |
| [35 , 40) | 0.0035 | 0.0035 | 0.0051 | 0.0065 | 0.0044 | 0.0044 | 0.0069 | 0.0069 |
| [40 , 45) | 0.0036 | 0.0049 | 0.0049 | 0.0077 | 0.0036 | 0.0036 | 0.0062 | 0.0116 |
| [45 , 50) | 0.0036 | 0.0046 | 0.0044 | 0.0068 | 0.0044 | 0.0051 | 0.0077 | 0.008 |
| [50 , 55) | 0.0032 | 0.0048 | 0.0055 | 0.0054 | 0.004 | 0.0047 | 0.0073 | 0.0058 |
| [55 , 60) | 0.0038 | 0.0049 | 0.0051 | 0.0066 | 0.0046 | 0.0044 | 0.007 | 0.0131 |
| [60 , 65) | 0.0046 | 0.0054 | 0.0056 | 0.009 | 0.0054 | 0.009 | 0.0117 | 0.0078 |
| [65 , 70) | 0.0049 | 0.0061 | 0.0083 | 0.0116 | 0.0057 | 0.0093 | 0.0119 | 0.0151 |
| **Female** | | | |  |  |  |  |  |
| Ages: [15 , 20) | 0.0013 | 0.0013 | 0.0021 | 0.0021 | 0.002 | 0.002 | 0.002 | 0.002 |
| [20 , 25) | 0.0013 | 0.0013 | 0.0023 | 0.0023 | 0.002 | 0.002 | 0.002 | 0.002 |
| [25 , 30) | 0.0013 | 0.0013 | 0.0023 | 0.0023 | 0.0028 | 0.0028 | 0.0036 | 0.0036 |
| [30 , 35) | 0.0014 | 0.0014 | 0.0022 | 0.0022 | 0.002 | 0.002 | 0.0027 | 0.0027 |
| [35 , 40) | 0.0014 | 0.0014 | 0.0023 | 0.0051 | 0.0026 | 0.0026 | 0.0046 | 0.0046 |
| [40 , 45) | 0.0014 | 0.0014 | 0.0024 | 0.0051 | 0.0033 | 0.0033 | 0.003 | 0.0048 |
| [45 , 50) | 0.0015 | 0.0015 | 0.0026 | 0.0062 | 0.0037 | 0.0037 | 0.0037 | 0.0094 |
| [50 , 55) | 0.0015 | 0.0015 | 0.0025 | 0.0041 | 0.002 | 0.003 | 0.0041 | 0.0063 |
| [55 , 60) | 0.0016 | 0.003 | 0.0027 | 0.0039 | 0.003 | 0.0062 | 0.0072 | 0.0075 |
| [60 , 65) | 0.0023 | 0.0028 | 0.0042 | 0.0059 | 0.0041 | 0.0044 | 0.0051 | 0.0093 |
| [65 , 70) | 0.0037 | 0.0041 | 0.005 | 0.0079 | 0.0055 | 0.0073 | 0.008 | 0.0146 |

Table 10: Adjusted estimates of annual risks for CHD by risk category, sex and age for the total population of Kenya. Each cell represent the average value of annual risk for the corresponding CVD event among all individuals falling with that strata. missing observations are adjusted by taking the average difference between the cells immediately to the left and above such that the values are increasing from left-to-right (for higher risk categories) and top-to-bottom (for older ages). Each panel is highlighted based on cells’ values ranging from lowest (blue) to highest (pink).

| **Risk category** | | | | | | | | | |
| --- | --- | --- | --- | --- | --- | --- | --- | --- | --- |
| **Male** | **1** | **2** | **3** | **4** | **5** | **6** | **7** | **8** |  |
| Ages: [15 , 20) | 0.001 | 0.001 | 0.001 | 0.001 | 0.001 | 0.001 | 0.001 | 0.001 |  |
| [20 , 25) | 0.001 | 0.001 | 0.001 | 0.001 | 0.001 | 0.001 | 0.001 | 0.001 |  |
| [25 , 30) | 0.001 | 0.001 | 0.0011 | 0.0011 | 0.001 | 0.001 | 0.001 | 0.001 |  |
| [30 , 35) | 0.001 | 0.001 | 0.001 | 0.001 | 0.001 | 0.001 | 0.002 | 0.002 |  |
| [35 , 40) | 0.0014 | 0.0014 | 0.0014 | 0.0085 | 0.001 | 0.001 | 0.002 | 0.002 |  |
| [40 , 45) | 0.0013 | 0.006 | 0.0015 | 0.0026 | 0.001 | 0.001 | 0.001 | 0.003 |  |
| [45 , 50) | 0.0024 | 0.0071 | 0.0024 | 0.006 | 0.0013 | 0.0105 | 0.0105 | 0.0048 |  |
| [50 , 55) | 0.003 | 0.0066 | 0.0033 | 0.0076 | 0.0019 | 0.0111 | 0.0111 | 0.0047 |  |
| [55 , 60) | 0.0044 | 0.0106 | 0.0043 | 0.0084 | 0.0033 | 0.0083 | 0.0083 | 0.0111 |  |
| [60 , 65) | 0.006 | 0.0094 | 0.0094 | 0.0105 | 0.005 | 0.0135 | 0.0135 | 0.0116 |  |
| [65 , 70) | 0.0073 | 0.0106 | 0.0083 | 0.0137 | 0.0062 | 0.0148 | 0.0148 | 0.0122 |  |
| **Female** | | | |  |  |  |  |  |  |
| Ages: [15 , 20) | 0 | 0 | 0 | 0 | 0 | 0 | 0 | 0 |  |
| [20 , 25) | 0 | 0 | 0 | 0 | 0 | 0 | 0 | 0 |  |
| [25 , 30) | 0 | 0 | 0 | 0 | 0 | 0 | 0 | 0 |  |
| [30 , 35) | 0 | 0 | 0 | 0 | 0 | 0 | 0 | 0 |  |
| [35 , 40) | 1E-05 | 1E-05 | 6E-05 | 0.001 | 0 | 0 | 0 | 0 |  |
| [40 , 45) | 1E-05 | 1E-05 | 0.0002 | 0.0007 | 0 | 0 | 0 | 0.0007 |  |
| [45 , 50) | 0.0002 | 0.0002 | 0.0006 | 0.0013 | 0 | 0 | 0.0003 | 0.002 |  |
| [50 , 55) | 0.0005 | 0.0005 | 0.0012 | 0.0032 | 0 | 0.002 | 0.001 | 0.0021 |  |
| [55 , 60) | 0.0012 | 0.003 | 0.0016 | 0.0041 | 0.001 | 0.0062 | 0.0052 | 0.004 |  |
| [60 , 65) | 0.0014 | 0.0041 | 0.0022 | 0.0043 | 0.001 | 0.0024 | 0.002 | 0.0035 |  |
| [65 , 70) | 0.0026 | 0.0044 | 0.003 | 0.0059 | 0.0021 | 0.0031 | 0.0027 | 0.0082 |  |

## Coupling Spectrum with the HIV/CVD microsimulation

Figure 1.C in the main text shows the relationships and flow of information between Spectrum and the HIV/CVD microsimulation. Annual outputs are collected from the Spectrum model from 2018 to 2033 and are used to inform the HIV/NCD microsimulations in corresponding years. Each microsimulation starts at the end of year 2018, and simulation experiments are carried out from year 2019 to 2033 as shown in Fig1.D.

The initial population is informed via outputs from the Spectrum model in year 2018 in terms of population size, HIV prevalence and ART coverage by strata of sex and age (five-year categories).

Annual HIV dynamics are initially modeled via Spectrum from 2019 to 2033, for both the baseline and intervention scenario, and projected outputs are used to inform the corresponding microsimulations. Specifically, the number of new HIV infections in the microsimulation in each year (from 2019 to 2033) is determined by the corresponding projected HIV incidence in Spectrum for that year. Once the number of incident HIV infections within each age/sex stratum is determined, new HIV transmissions are incorporated into the microsimulation by drawing a random sample of HIV-uninfected individuals in each age/sex stratum-strata and moving them to an HIV-infected state. ART coverage is calibrated to the reported proportion of HIV-infected individuals receiving ART in each age/sex stratum-strata from Spectrum, and is updated on an annual basis. HIV deaths are similarly calibrated to projected data from Spectrum in each year, and these deaths are modeled to occur randomly among the HIV-infected population in the microsimulation.

CVD dynamics are modeled directly at an individual level and on an annual basis as described above.

Finally, the additional probability of death due to non-HIV/non-CVD causes is estimated by subtracting the simulated number of HIV and CVD deaths from Spectrum’s projected estimate of all-cause mortality in each year (divided, and dividing by the appropriate population size). These deaths are modeled to occur in the microsimulation at random within each age/sex stratum in each year.

**Choice of time step:** The microsimulation model runs in discrete timesteps representing one year. The choice of simulation timestep was selected to correspond to the annual timesteps in the Spectrum model. The projected data on population demography and HIV epidemiology were reported on an annual basis by Spectrum and were used to update the HIV-NCD model on an annual basis. In absence of data on HIV epidemiology within a smaller timestep (e.g., monthly) from the Spectrum model, we were not able to test any shorter timesteps in our analysis.

# Experimental scenarios

The intervention scenario represents a community-wide outreach program, similar to project SEARCH (https://www.searchendaids.com) for screening and treatment of HIV, hypertension and diabetes. The intervention will roll out for 5 years and targets 20% of population on an annual basis. We assume that the targeted populations are geographically independent, such any individual can be targeted only once. We further assume 90% successful testing among eligible population (15 years and older) based on recent results from project SEARCH Among those screened, ART uptake is modeled at 73% for those HIV-infected individuals with no previous or current ART use^[7-9]^.

We apply the SPECTRUM model to implement the HIV screening and ART uptake intervention. For this purpose, we estimate the expected increase in number of ART initiators throughout the intervention scenario and model the gradual increase in ART coverage during the intervention period (2019 to 2023). Assuming a fix ART coverage after the intervention ends, we follow the population for the next 10 years until year 2033. This intervention scenario is compared against a baseline scenario in which ART coverage was maintained at 2017 levels until 2033.

We further model the impact of NCD component by screening individuals for hypertension and diabetes and offering treatment to those identified with either or both conditions. Based on our underlying risk categorization, this translates to individuals in risk category 3 or higher (who are 15 years and older) in our model. Similar to previous studies^[2]^, we assume a 50% uptake for diabetes and hypertension treatment among eligible population, and vary this value in sensitivity analysis. The impact of medication management is determined by adjusting the risk of acute CVD events by the anticipated percentage decline in future CHD and stroke events. To determine the impact of hypertension treatment, we estimated the average decrease in risk of CHD and stroke events based on reduction in blood pressure as reported by hypertension treatment trials. We specifically focused on a study by Ettehad et al. (2015) that estimates the impact of 10 mm Hg reduction in systolic blood pressure on the relative risk (RR) of CHD and stroke in presences or absence of diabetes^[10]^. Furthermore, we estimate the impact of Metformin, as a first line treatment for diabetes, on CVD outcomes. In a meta-analysis of randomized clinical trials, Lamanna et al. (2010) suggest a significant benefit from Metformin compared to no therapy for CVD events^[11]^. Finally, in absence of data on combined impact of treatment for hypertension and diabetes at the same time, we adopt a simplifying assumption that the impact is independent and therefore multiplicative (Table 11). In addition, we assumed that once individuals are linked to consistent care for hypertension or diabetes, they will be screened and treated for the other NCD if it were to develop (e.g., A non-diabetic person receiving hypertension treatment in year 2020 is considered actively in care, so if he/she develop diabetes at a later year, he/she will be eligible to receive diabetes treatment.)

Table 11: Estimated impact of hypertension and diabetes treatment on risk of CHD and stroke.

| 1. Data from the literature | Reduction in risk of CHD  RR [95% CI] | Reduction in risk of Stroke  RR [95% CI] |
| --- | --- | --- |
| Hypertension treatment ^[10]^   - Diabetes - No Diabetes | 0.88 [0.8 – 0.97]  0.77 [0.70 – 0.86] | 0.74 [0.65 – 0.84]  0.74 [0.67 – 0.81] |
| Diabetes treatment ^[11]^ | - 1. 0.64 – 0.98] | |
| 1. Modeled impact of treatment | **Reduction in risk of CHD** | **Reduction in risk of Stroke** |
| Combined impact of treatment for hypertension and diabetes | 0.88 * 0.79 =0.69 | 0.74 * 0.79 =0.58 |
| Single treatment for hypertension | 0.77 | 0.74 |
| Single treatment for diabetes | 0.79 | 0.79 |

# Cost-effectiveness

The cost-effectiveness analyses of the modeling scenarios are based on the resources required to deliver the intervention, the impact of the intervention on healthcare utilization, and the impact of the intervention on death and disability expressed as disability-adjusted life years (DALYs). From the resources required and healthcare utilization (valued in 2018 USD) we estimate total costs from the healthcare perspective and DALYs averted for the status quo and intervention scenarios. From these values we calculate the incremental cost, the incremental effectiveness, and the incremental cost-effectiveness ratio (incremental cost per incremental DALY averted) in the intervention scenario relative to the status quo scenario. Interventions are often considered highly cost effective in a given country if the net cost per DALY averted is less than the per-capita gross domestic product (GDP). In conjunction with this commonly used standard, it is essential to be cognizant of affordability, feasibility, and equity when evaluating these scenarios.

## Cost analysis

For status quo and intervention scenarios, we established costs associated with community-based screening of HIV, hypertension, and diabetes; treatment costs associated with routine care for HIV, hypertension, and diabetes; and treatment costs subsequent to CVD events. These cost estimates were then applied to the number of individuals projected to receive each type of care in each year of the simulated scenarios.

Cost estimates were identified through reviews of published and grey literature and chosen based on discussions among the study investigators. Preferences were given to estimates that we believed would be closest to expected in the intervention scenarios. As the intervention is based directly on the SEARCH study protocol, we used cost estimates from SEARCH whenever possible. This included cost estimates from cost analyses of community-based screening for HIV and NCDs and ART treatment observed in the SEARCH study ^[12, 13]^. Chang et al. ^[13]^ provide a cost analysis of hybrid mobile multi-disease testing conducted in Western Kenya and Uganda as part of the SEARCH study. Reported HIV related costs were $20.5 (2014 USD) per person, with a range of $18.9 to $21.6, and with the majority of costs related to the community health campaign. The reported joint cost for hypertension and diabetes screening was $1.16, with no range reported. This value reflects the marginal cost of screening given the expenditure of HIV-related costs. The authors also provide a breakdown of resources required and their costs in an appendix. In 2018 USD, the cost of HIV screening was $21.50, and the marginal cost of hypertension and diabetes testing was $1.22.

The cost of antiretroviral treatment was also based on the SEARCH study. Shade et al. (2018) ^[14]^ measured costs of HIV care among SEARCH study participants at 17 health care clinics in Uganda and Kenya. The observed cost of care in Kenya was $285.52 per person year (in 2016 USD), with the majority of cost attributable to ART and viral load testing. With lower ART costs, centralized viral load testing, and personnel costs commensurate with those observed in the Kenya Ministry of Health, the authors estimate a possible lower per person year cost of $163. For the present cost-effectiveness analysis, we used the observed cost of $285.52 ($297.51 in 2018 USD).

Costs of standard hypertension and diabetes care and CVD-related events and health states were based on those estimated from public and semi-public health care facilities in Kenya ^[15]^, and that were subsequently used in a modeling study of the cost-effectiveness of stratified care for CVD in Kenya ^[2]^. Subramanian et al. (2018) ^[15]^ used patient payment data from Kenyatta National Hospital to estimate costs for hypertension and diabetes treatment at a public facility and used an ingredient-based approach to estimate treatment costs at MSF Belgium's Kibera South Health Center, a quasi-public facility. These costs included clinic visit cost, diagnostic tests, procedures, and medications. (Costs associated with major complications were assessed and categorized separately, as described below.) Estimated yearly cost (in 2017 USD) for hypertension management ranged from $25.64 for a 1-drug regimen to $110.33 for a 4-drug regimen, with a cost of $159.36 for a treatment-resistant regimen. Using a weighted average based on the estimated distribution of treatment regimens, the expected annual cost of hypertension management was $75.93 ($77.65 in 2018 USD). Costs for diabetes care included oral medication only, insulin only, and both insulin and oral medication regimens. Using a weighted average based on the estimated distribution of treatment regimens, the expected annual cost of diabetes treatment was $182.61 ($186.73 in 2018 USD). As we expect that health care would be provided through public or semi-public facilities, we did not incorporate estimates of the cost of hypertension and diabetes care in private health care facilities from the same study.

Costs for CVD-related events were also derived from the same study ^[15]^, again using estimates for public and semi-public health care facilities. These included costs for stroke, myocardial infarction, angina, and heart failure secondary to hypertension. Costs not derived from this study include annual costs of care post-stroke and post-CHD. As was the case in the modeling study by Subramanian et al. ^[2]^, we used estimates from a 2014 cost-effectiveness analysis of a hypertension intervention in South Africa by Gaziano et al. ^[16]^ for estimates of annual post-stroke care ($993.44 in 2018 USD) and annual post-CHD care ($331.15 in 2018 USD). In this same issue of *JAIS*, Sandro et al. ^[17]^ provide cost estimates of non-acute CVD care, including stroke and ischemic heart disease, for Uganda of $200 in 2017 USD, with a 95% confidence interval of $74 to $340. While the estimate we use for post-CHD care is within this range, the estimate for post-stroke care is substantially higher. This indicates a need for additional cost analyses of CVD-related care in Kenya.

## Disability-adjusted life years (DALYs)

For each scenario, DALYs were estimated as years lost to HIV-related or CVD-related disability (YLD) and years of life lost (YLL) to HIV-related or CVD-related premature mortality. YLD were calculated for each year and each HIV-related or CVD-related health state using disability weights from the 2017 Global Burden of Disease study ^[18]^. Not all health states from our model mapped directly to specific health states in the Global Burden of Disease Study. For example, the latter includes different levels of impairment following stroke, whereas our models did not distinguish levels of impairment. In such instances we chose DWs corresponding to “moderate” health states. DWs for comorbid states are based on the approach of Vos et al. (2012) ^[19]^. The comorbid DW was estimated as one minus the product of the complements of the disability weights for the individual conditions):

$${DW}_{c}=1-\prod_{k=1}^{j} \left( 1-{DW}_{k} \right)$$

where *j* represents the number of health states reflected in the comorbid health state *c*.

For a given year, the YLD across all relevant health states is given by:

$${YLD}_{t}=\sum_{k=1}^{j} n_{tk}*{DW}_{k}$$

where *t* indexes year, *k* is one of *j* health states, $n_{tk}$ is the number of individuals in health state *k* at time *t*, and ${DW}_{k}$ is the disability weight associated with health state *k*.

YLL were calculated for each year, age group, and sex. YLL were defined as the difference between minimum life expectancy and age of HIV- or CVD-related death, with future YLL discounted at 3% per annum. For estimates of minimum life expectancy, we used life expectancy tables for Kenya in 2017 from the Global Burden of Disease study ^[20]^. Specifically, we used life expectancy in the absence of HIV and in the absence of population shocks. Consistent with GBD approach, age discounting was not used. For a given death, the discounted YLL is given by:

$${YLL}_{i}=\frac{1-e^{-r*{LE}_{i}}}{\left( 1-r \right)}$$

where *r* is the discount rate (0.03 in the base case) and ${LE}_{i}$ is life expectancy for person *i*.

Across *m* individuals experiencing death at time *t*, the total YLL is estimated as follow:

$${YLL}_{t}=\sum_{i=1}^{m_{t}} {YLL}_{ti}$$

YLL associated with a death were assigned to the year in which the death occurred. Thus, DALYs for a given year incorporated YLD for health states experienced in that year and discounted future YLL for deaths occurring in that year.

## Incremental cost-effectiveness

The differences between the status quo and intervention scenarios for total costs and total DALYs across all years provided the estimates of incremental costs and incremental DALYs averted, and the ratio of these differences, in turn, provided the incremental cost-effectiveness ratio. The cost-effectiveness ratio was reported at the end of simulation period (year 2033) and was assessed against a threshold of $2,010 corresponding to per-capita gross domestic product (GDP) in Kenya in 2019 ^[21]^, as well as other thresholds (at increments of $500 per DALY) reflect other possible willingness to pay thresholds in Kenya.

# Results

## Initial population summary

Table 12 shows the population summary characteristics for the national and regional models in year 2018. Population size and HIV-related measures are provided by the Spectrum models and are used to inform the initial simulated populations in year 2018 in the corresponding HIV/NCD microsimulations. Prevalence of hypertension and diabetes is estimated using the 2015 STEPwise data.

Table 12: Population summary characteristics for the national and regional models.

| **Region** | **Measure** | **Total** | **Male** | **Female** |
| --- | --- | --- | --- | --- |
| **National** | **Population size** | 50,994,588 | 25,379,064 | 25,615,526 |
|  | **Number living with HIV** | 1,707,484 | 612,453 | 1,095,031 |
|  | **New infections** | 46,438 | 16,419 | 30,020 |
|  | **Number receiving ART** | 1,115,425 | 362,666 | 752,758 |
|  | **HIV prevalence %** | 3.35% | 2.41% | 4.27% |
|  | **HIV incidence per 1,000 person year** | 0.911 | 0.647 | 1.172 |
|  | **ART coverage %** | 65.33% | 59.22% | 68.74% |
|  | **Hypertension prevalence** | 23.82% | 25.06% | 22.64% |
|  | **Diabetes prevalence** | 1.92% | 1.50% | 2.32% |
| **Nairobi** | **Population size** | 4,937,717 | 2,411,524 | 2,526,193 |
|  | **Number living with HIV** | 208,929 | 76,235 | 132,694 |
|  | **New infections** | 4,866 | 1,875 | 2,992 |
|  | **Number receiving ART** | 149,272 | 50,002 | 99,270 |
|  | **HIV prevalence %** | 4.23% | 3.16% | 5.25% |
|  | **HIV incidence per 1,000 person year** | 0.985 | 0.778 | 1.184 |
|  | **ART coverage %** | 71.45% | 65.59% | 74.81% |
|  | **Hypertension prevalence** | 13.59% | 15.89% | 11.12% |
|  | **Diabetes prevalence** | 3.27% | 2.42% | 4.23% |
| **Central** | **Population size** | 5,125,269 | 2,562,856 | 2,562,413 |
|  | **Number living with HIV** | 135,658 | 47,818 | 87,840 |
|  | **New infections** | 3,256 | 1,113 | 2,143 |
|  | **Number receiving ART** | 96,871 | 29,280 | 67,591 |
|  | **HIV prevalence %** | 2.65% | 1.87% | 3.43% |
|  | **HIV incidence per 1,000 person year** | 0.635 | 0.434 | 0.836 |
|  | **ART coverage %** | 71.41% | 61.23% | 76.95% |
|  | **Hypertension prevalence** | 36.14% | 34.25% | 37.99% |
|  | **Diabetes prevalence** | 2.55% | 0.97% | 4.13% |
| **Coast** | **Population size** | 4,348,662 | 2,176,610 | 2,172,052 |
|  | **Number living with HIV** | 138,686 | 48,253 | 90,433 |
|  | **New infections** | 4,367 | 1,585 | 2,781 |
|  | **Number receiving ART** | 82,139 | 26,164 | 55,975 |
|  | **HIV prevalence %** | 3.19% | 2.22% | 4.16% |
|  | **HIV incidence per 1,000 person year** | 1.004 | 0.728 | 1.280 |
|  | **ART coverage %** | 59.23% | 54.22% | 61.90% |
|  | **Hypertension prevalence** | 19.23% | 21.43% | 16.59% |
|  | **Diabetes prevalence** | 2.03% | 3.08% | 0.69% |

## Intervention outcomes

Table 13: Projected burden of untreated hypertension and diabetes in year 2018 (baseline) and year 2033, comparing baseline with the intervention scenario. Values represent reported as median values [95% uncertainty ranges] across 2,000 random simulations.

|  | **National** | **Nairobi region** | **Central region** | **Coast region** |
| --- | --- | --- | --- | --- |
| **Model initiation, Dec 2018** | | | | |
| **Untreated hypertension** | | | | |
| **Prevalence (%)** | 14.95  [14.92 - 14.98] | 9.33  [9.31 - 9.36] | 26.23  [26.2 - 26.26] | 12.08  [12.06 - 12.11] |
| **Frequency (thousand)** | 7,624.18  [7,610.4 – 7,637.95] | 460.82  [459.7 - 461.96] | 1,344.32  [1,342.67 – 1,345.9] | 525.51  [524.32 - 526.76] |
| **Untreated Diabetes** | | | | |
| **Prevalence (%)** | 1.36  [1.35 - 1.37] | 2.67  [2.65 - 2.68] | 2.01  [2 - 2.02] | 1.19  [1.18 - 1.2] |
| **Frequency (thousand)** | 692.4  [687.34 - 697.31] | 131.66  [131.05 - 132.3] | 103.15  [102.59 - 103.75] | 51.81  [51.39 - 52.24] |
| **Baseline scenario, year 2033** | | | | |
| **Untreated hypertension** | | | | |
| **Prevalence (%)** | 32.43  [32.4 - 32.45] | 25.94  [25.91 - 25.97] | 47.89  [47.86 - 47.91] | 26.41  [26.39 - 26.44] |
| **Frequency (thousand)** | 23,000.6 [  22,984.19 - 23018.4] | 17,19.23  [1,717.48 – 1,721] | 3,011.72  [3,010.08 – 3,013.35] | 1,615.8  [1,614.18 – 1,617.42] |
| **Untreated Diabetes** | | | | |
| **Prevalence (%)** | 4.27  [4.25 - 4.28] | 9.75  [9.73 - 9.77] | 6.05  [6.04 - 6.07] | 3.4  [3.39 - 3.42] |
| **Frequency (thousand)** | 3,026.46  [3,017.42 – 3,035.59] | 646.28  [645.15 - 647.47] | 380.59  [379.63 - 381.64] | 208.25  [207.46 - 209.06] |
| **Intervention scenario, year 2033** | | | | |
| **Untreated hypertension** | | | | |
| **Prevalence (%)** | 27.51  [27.48 - 27.53] | 22.61  [22.58 - 22.63] | 38.73  [38.7 - 38.76] | 22.58  [22.55 - 22.61] |
| **Frequency (thousand)** | 19,613.63  [19,593.6 – 19,633.8] | 1,506.82  [1,505.19 – 1,508.44] | 2,447.46  [2,445.39 – 2,449.44] | 1,387.08  [1,385.34 – 1,388.91] |
| **Untreated Diabetes** | | | | |
| **Prevalence (%)** | 3.42  [3.41 - 3.44] | 8.43  [8.41 - 8.45] | 4.43  [4.42 - 4.45] | 2.79  [2.78 - 2.8] |
| **Frequency (thousand)** | 2,440.67  [2,431.94 – 2,449.61] | 562.05  [560.81 - 563.25] | 280  [279.15 - 280.91] | 171.45  [170.7 - 172.2] |

# References

1. Stover J, Brown T, Puckett R, Peerapatanapokin W. **Updates to the Spectrum/Estimations and Projections Package model for estimating trends and current values for key HIV indicators**. *Aids* 2017; 31 Suppl 1:S5-S11.

2. Subramanian S, Hilscher R, Gakunga R, Munoz B, Ogola E. **Cost-effectiveness of risk stratified medication management for reducing premature cardiovascular mortality in Kenya**. *PLoS One* 2019; 14(6):e0218256.

3. **Kenya stepwise survey for non communicable diseases risk factors 2015 report**. In; 2015.

4. D'Agostino RB, Sr., Vasan RS, Pencina MJ, Wolf PA, Cobain M, Massaro JM, et al. **General cardiovascular risk profile for use in primary care: the Framingham Heart Study**. *Circulation* 2008; 117(6):743-753.

5. D'Agostino RB, Wolf PA, Belanger AJ, Kannel WB. **Stroke risk profile: adjustment for antihypertensive medication. The Framingham Study**. *Stroke* 1994; 25(1):40-43.

6. Expert Panel on Detection E, Treatment of High Blood Cholesterol in A. **Executive Summary of The Third Report of The National Cholesterol Education Program (NCEP) Expert Panel on Detection, Evaluation, And Treatment of High Blood Cholesterol In Adults (Adult Treatment Panel III)**. *JAMA* 2001; 285(19):2486-2497.

7. Chamie G, Clark TD, Kabami J, Kadede K, Ssemmondo E, Steinfeld R, et al. **A hybrid mobile approach for population-wide HIV testing in rural east Africa: an observational study**. *The lancet HIV* 2016; 3(3):e111-119.

8. Chamie G, Kwarisiima D, Clark TD, Kabami J, Jain V, Geng E, et al. **Leveraging rapid community-based HIV testing campaigns for non-communicable diseases in rural Uganda**. *PLoS One* 2012; 7(8):e43400.

9. Chamie G, Kwarisiima D, Clark TD, Kabami J, Jain V, Geng E, et al. **Uptake of community-based HIV testing during a multi-disease health campaign in rural Uganda**. *PLoS One* 2014; 9(1):e84317.

10. Ettehad D, Emdin CA, Kiran A, Anderson SG, Callender T, Emberson J, et al. **Blood pressure lowering for prevention of cardiovascular disease and death: a systematic review and meta-analysis**. *Lancet* 2016; 387(10022):957-967.

11. Lamanna C, Monami M, Marchionni N, Mannucci E. **Effect of metformin on cardiovascular events and mortality: a meta-analysis of randomized clinical trials**. *Diabetes Obes Metab* 2011; 13(3):221-228.

12. Jain V, Chang W, Byonanebye DM, Owaraganise A, Twinomuhwezi E, Amanyire G, et al. **Estimated Costs for Delivery of HIV Antiretroviral Therapy to Individuals with CD4+ T-Cell Counts >350 cells/uL in Rural Uganda**. *PLoS One* 2015; 10(12):e0143433.

13. Chang W, Chamie G, Mwai D, Clark TD, Thirumurthy H, Charlebois ED, et al. **Implementation and Operational Research: Cost and Efficiency of a Hybrid Mobile Multidisease Testing Approach With High HIV Testing Coverage in East Africa**. *Journal of acquired immune deficiency syndromes (1999)* 2016; 73(3):e39-e45.

14. Shade SB, Osmand T, Luo A, Aine R, Assurah E, Mwebaza B, et al. **Costs of streamlined HIV care delivery in rural Ugandan and Kenyan clinics in the SEARCH Studys**. *AIDS (London, England)* 2018; 32(15):2179-2188.

15. Subramanian S, Gakunga R, Kibachio J, Gathecha G, Edwards P, Ogola E, et al. **Cost and affordability of non-communicable disease screening, diagnosis and treatment in Kenya: Patient payments in the private and public sectors**. *PLOS ONE* 2018; 13(1):e0190113.

16. Gaziano TA, Bertram M, Tollman SM, Hofman KJ. **Hypertension education and adherence in South Africa: a cost-effectiveness analysis of community health workers**. *BMC Public Health* 2014; 14(1):240.

17. Sando D, Kintu A, Okello S, Kawungezi P, Guwatudde D, Mutungi G, et al. **Cost-effectiveness analysis of integrating screening and treatment of selected non-communicable disease risk factors into HIV/AIDS treatment in Uganda**. *Journal of the International AIDS Society* 2020; 23(2):e2XXX.

18. Global Burden of Disease Collaborative Network. **Global Burden of Disease Study 2017 (GBD 2017) Disability Weights**. In. Seattle, WA: Institute for Health Metrics and Evaluation (IHME); 2018.

19. Vos T, Flaxman AD, Naghavi M, Lozano R, Michaud C, Ezzati M, et al. **Years lived with disability (YLDs) for 1160 sequelae of 289 diseases and injuries 1990-2010: a systematic analysis for the Global Burden of Disease Study 2010**. *Lancet* 2012; 380(9859):2163-2196.

20. Global Burden of Disease Collaborative Network. **Global Burden of Disease Study 2017 (GBD 2017) Life Tables 1950-2017**. In. Seattle, WA: Institute for Health Metrics and Evaluation (IHME); 2018.

21. International Monetary Fund (IMF). **Report for Selected Countries and Subjects**. In; 2019.
